# Photonic time crystals assisted by quasi-bound states in the continuum

**DOI:** 10.1126/sciadv.aed4055

**Published:** 2026-08-12

**Authors:** Puneet Garg, Evangelos Almpanis, Leander Zimmer, Jan David Fischbach, Xuchen Wang, Mohammad S. Mirmoosa, Markus Nyman, Nikolaos Stefanou, Nikolaos Papanikolaou, Viktar Asadchy, Carsten Rockstuhl

**Affiliations:** ^1^ Institute of Theoretical Solid State Physics, Karlsruhe Institute of Technology, Kaiserstrasse 12, Karlsruhe 76131, Germany.; ^2^ Section of Condensed Matter Physics, National and Kapodistrian University of Athens, Panepistimioupolis, Athens 15784, Greece.; ^3^ Institute of Nanoscience and Nanotechnology, NCSR “Demokritos”, Patriarchou Gregoriou and Neapoleos Str., Ag. Paraskevi, Athens 15310, Greece.; ^4^ Institute of Nanotechnology, Karlsruhe Institute of Technology, Kaiserstrasse 12, Karlsruhe 76131, Germany.; ^5^ College of Physics and Optoelectronic Engineering, Harbin Engineering University, Nantong Street 145, Harbin 150001, China.; ^6^ Department of Physics and Mathematics, University of Eastern Finland, Yliopistokatu 7, Joensuu 80101, Finland.; ^7^ Department of Electronics and Nanoengineering, Aalto University, Maarintie 8, Espoo 02150, Finland.

## Abstract

Photonic time crystals (PTCs) are characterized by the rapid modulation of the material properties in time, causing a momentum bandgap for light. However, the observation of these bandgaps at optical frequencies remains elusive as the necessary temporal modulation amplitudes to show notable momentum bandgaps are relatively high, inaccessible with available materials. While it has been known that structuring PTCs at the subwavelength scale can improve the bandgap size, we push this concept to the extreme by leveraging the nanophotonic toolbox. Specifically, we demonstrate that structures composed of scatterers supporting quasi-bound states in the continuum can substantially reduce the required modulation amplitudes by enhancing the interaction time between light and time-varying matter. This allows us to observe noticeable momentum bandgaps despite the weak temporal modulation. Our approach bridges the concepts of bound states in the continuum and time-varying metamaterials, paving the way toward realizable PTCs at optical frequencies.

## INTRODUCTION

Recently, there has been a surge of interest in the physics of time-varying photonic media (*1*–*13*). In particular, photonic time crystals (PTCs), i.e., materials characterized by periodic modulation of the permittivity in time, have been at the forefront of this research domain (*14*–*19*). The salient property of PTCs is that they exhibit momentum bandgaps. Inside these bandgaps, eigenmodes exist with complex frequencies for a certain range of wave numbers. Depending on the sign of the imaginary part, the complex frequency modes amplify the incident radiation that couples to them (*15*, *20*). PTCs show promise for intensifying light-matter interactions, much like spatial photonic crystals enhance light confinement (*21*, *22*). Applications of momentum bandgaps in PTCs include amplified spontaneous emission (*23*, *24*), subluminal Cherenkov radiation (*25*), and temporal localization effects (*26*–*29*), among many others.

Although PTCs have been recently experimentally demonstrated at microwave frequencies thanks to the availability of fast variable electronic and radio frequency components (*30*–*32*), their realization at even higher frequencies remains a major challenge. Achieving the required temporal modulation demands extremely fast material changes, typically at twice the oscillation frequency of the probing light, and a relative permittivity change close to unity when considering a bulk (i.e., a spatially homogeneous) PTC (*15*, *33*). The optical modulation of transparent conducting oxides (TCOs) by an external pump has emerged as a viable path to study effects related to time-varying media. In particular, indium tin oxide (ITO) and aluminum-doped zinc oxide (AZO) offer relative permittivity changes of up to 100% in their epsilon-near-zero (ENZ) frequency regime (*34*–*38*). The permittivity modulation in TCOs can be achieved using hot electron nonlinearities (*38*) as well as third-order nonlinearities (*10*). However, achieving such modulation requires extremely high pump intensities (*33*). Combined with substantial material dissipation, these requirements pose a risk of thermal damage, making practical implementation of PTCs with TCOs highly challenging (*39*). Moreover, achieving a “pure PTC” regime using time-varying bulk TCO platforms may prove difficult (*33*, *40*).

A loophole to achieve a PTC not obscured by other effects is to use third-order nonlinearity in transparent materials, such as Si, Ge, or GaAs (*33*, *41*). For example, the effects of third-order nonlinearity (under the assumption of an undepleted pump) can be modeled using linear coupled-mode equations of a spatially uniform material with time-varying permittivity. The modulation frequency in this case can be extremely high, virtually unlimited (*33*). However, in low-loss materials, nonlinear effects are inherently weak (*42*), restricting the relative permittivity change to a maximum of not more than 1%. This tiny modulation amplitude is insufficient to observe the main features of PTCs. Inevitable scattering losses in realistic material systems and other imperfections may easily obstruct the weak signal amplification. Nevertheless, a recent theoretical study demonstrated that momentum bandgaps in low-loss PTCs can be expanded even with weak permittivity modulation amplitudes by leveraging structural resonances in a metasurface (*43*). The proposed metasurface comprised an array of subwavelength resonant silicon spheres that support dipolar Mie resonances. However, this design suffers from limitations in that the resonances had low quality factors (*Q*-factors), which necessitate an operation close to the laser-induced damage threshold of realistic materials (*44*–*47*).

To overcome this limitation, we leverage here the concept of photonic quasi-bound states in the continuum (qBICs) and combine it with PTCs. Photonic qBICs are known to exhibit arbitrarily high *Q*-factors, limited only by material losses, finite-size effects, and fabrication tolerances (*48*–*51*). We demonstrate that metasurfaces made of nanoresonators supporting qBICs pave the way for the realization of practical PTCs using conventional materials and accounting for realistic losses. Specifically, we propose two metasurface geometries based on multilayered spherical and homogeneous cylindrical nanoresonators. The study of multilayered sphere-based metasurfaces sheds light on the theoretical aspects of expanding momentum bandgaps with qBICs. In particular, with this geometry, we study the effects of material-induced qBICs. Meanwhile, the cylinder-based metasurface demonstrates that noticeable momentum bandgaps are achievable despite realistic losses with reasonable designs. We show that these bandgaps are achieved while operating at pump intensities an order of magnitude below the laser-induced damage threshold. The cylinder-based metasurface uses geometry-induced qBICs. Therefore, for completeness, both structures are discussed. We also highlight that our work extends the Mie theory framework to time-varying metasurfaces made from multilayered spheres and finite-sized cylinders (*52*).

## RESULTS

### Concept

**Fig. 1.**
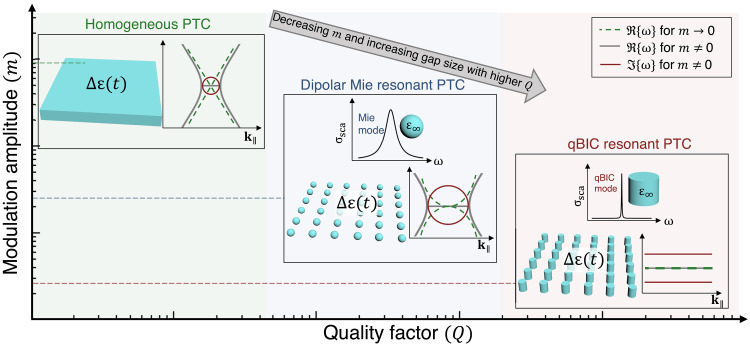
A conceptual illustration. This schematic discusses the modulation amplitude *m* of the material properties necessary to open momentum bandgaps as a function of the quality factor *Q* of the resonances supported by meta-atoms in various PTC geometries. We observe that, as *Q* increases, *m* decreases for increasing gap size. The illustration depicts a spatially homogeneous PTC (left column), a PTC constructed from meta-atoms that sustain the lowest-order Mie resonances (middle column), and the proposed PTC whose meta-atoms host high-*Q* qBICs (right column). The Mie and qBIC resonances of the static meta-atoms having background permittivity $\varepsilon_{\infty }$ are depicted in the plots of their respective scattering cross sections $\sigma_{\text{sca}}$. Here, Δε(t) represents the time-periodic background permittivity of the underlying media of the meta-atoms, ω is the frequency, and $k_{∥}$ is the in-plane wave vector of the eigenmodes of the PTCs. Besides the basic illustration of the geometry and resonances, the band structure is shown for zero (dotted green) and nonzero (solid gray and solid red) modulation amplitude *m*. Note that the high-*Q* resonance of an isolated static meta-atom leads to a strong localization of the field inside the meta-atom. This localization reduces the interaction between the neighboring meta-atoms when they are placed in a lattice, leading to reduced spatial dispersion in the lattice. Therefore, a flat band emerges in the band structure of the corresponding static metasurface.

We illustrate the proposed physical idea in Fig. 1. A conventional PTC is spatially homogeneous and consists of a medium whose material properties, such as the background permittivity Δε, change periodically in time, i.e., $\text{Δε}\left(t\right)=\varepsilon_{\infty }\left\{1+m\left[1+\text{cos}\left(\omega_{m}t\right)\right]\right\}$. Here, $\varepsilon_{\infty }$ is the background permittivity of the corresponding static medium, *m* is the modulation amplitude, and $\omega_{m}$ is the modulation frequency. Note that the background permittivity refers to the effective nonresonant contribution to total permittivity (see section S1) (*10*). We use the optical Kerr effect to account for the time-varying permittivity (*10*, *40*, *53*). In this scenario, the parameters of the permittivity modulation can be expressed as $m=\frac{\chi^{\left(3\right)}E_{p}^{2}}{2\varepsilon_{\infty }}$ and $\omega_{m}=2\omega_{p}$ (see sections S1 and S2). Here, $\chi^{\left(3\right)}$ is the third-order nonlinear susceptibility of the medium, and $E_{p}$ and $\omega_{p}$ are the real-valued field amplitude and the frequency of the pump wave, respectively. The dispersion relation of light in the bulk static medium in its low material loss regime is linear. The periodic time modulation, in a temporal empty lattice approximation (i.e., m→0 and $\omega_{m}\neq 0$), causes the periodic repetition of the dispersion relation in the frequency domain. Each branch is displaced by a multiple of $\omega_{m}$. These are the Floquet bands, and they cross at the edges of the frequency Brillouin zone. When switching on a finite amplitude for the time modulation (i.e., m≠0), a momentum bandgap opens around the crossing point. Within the momentum bandgap, the eigenfrequencies can have positive or negative imaginary parts. Assuming a time convention of $e^{-i\omega t}$, these two solutions exponentially grow or decay in time, respectively, upon excitation. However, the achievable momentum bandgap remains rather tiny, even for substantial modulation amplitudes (*33*).

To overcome this limitation, we can spatially structure the PTC and exploit basic photonic resonances supported by the structured material. A prototypical example of a photonic resonance is that of a Mie resonance. It occurs whenever a multipole moment sustained in the spatially localized scatterer is resonantly excited. Due to such a resonance, light remains trapped inside the scatterer for an extended duration, where it experiences the time-varying material properties.

When periodically arranging such Mie scatterers to form a metasurface, the bands in the static material experience a rather strong curvature as a consequence of the resonant modulation of the otherwise linear dispersion relation (see Fig. 1). Switching on the time modulation with a modulation frequency approximately twice the resonance frequency causes a repetition of the dispersion relation in the frequency domain, a crossing at the spectral location of the resonance, and a larger momentum bandgap. However, the approach has its limitations. The achievable *Q*-factors for ordinary Mie resonances remain rather low for the lowest-order multipole moments. These resonances couple efficiently to free space and, therefore, suffer from high radiation losses.

However, nanophotonics provides us with the tools to engineer scatterers with little to no radiation losses. These structures host bound states in the continuum (BICs) and are designed to have entirely suppressed radiative losses (*49*, *50*, *54*). Admittedly, the complete suppression of radiation losses is not desirable as we are still required to excite these resonances. Nevertheless, by deliberately breaking the symmetry or detuning the system incrementally from the BIC condition, we can deterministically adjust the *Q*-factor that the scatterer sustains. The resulting high-*Q* resonances are qBICs.

When placing these scatterers with qBICs in a metasurface lattice, a photonic flat band emerges for the stationary medium (see Fig. 1). Now, by choosing $\omega_{m}$ to be approximately twice the qBIC resonance frequency, in theory, an extremely wide momentum bandgap emerges for a tiny modulation amplitude. The crossing of the two flat bands, split by time modulation, eventually covers the entire Brillouin zone. By using our understanding of how to structure materials to induce resonances with ultrahigh *Q*-factors, we can pave the way for the realistic implementation of PTCs and their associated momentum bandgaps.

Note that, in this work, we exploit the flat bands of the metasurfaces to expand momentum bandgaps. The flatness of the bands is predominantly controlled by the *Q*-factors of the resonances of the isolated meta-atoms. Therefore, we optimize the individual meta-atoms (and not the metasurface) to host high-*Q* qBICs (see section S3).

In the following, we illustrate this concept with two different examples that achieve such scatterers with high *Q*-factors by leveraging different nanophotonic approaches. The first example concerns a metasurface made from ENZ-material–based multilayered spheres. The second example concerns a metasurface composed of germanium cylinders. In both examples, we capitalize on high-*Q* qBICs to show that the modulation amplitude required to obtain noticeable momentum bandgaps can be considerably reduced as compared to homogeneous PTCs and metasurfaces supporting ordinary Mie resonances.

### PTCs based on metasurfaces made from multilayered spheres

**Fig. 2.**
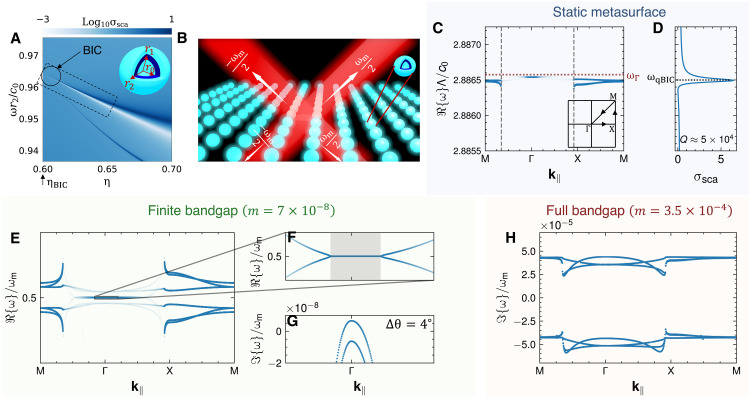
PTC based on multilayered sphere metasurface. (**A**) The normalized scattering cross section $\sigma_{\text{sca}}$ of the static multilayered sphere (see inset) as a function of the frequency ω and the geometry parameter $\eta =r_{1}/r_{2}$. The black circle marks the spectral location of the true BIC. Note that material losses are neglected here to clearly illustrate the effects. (**B**) A metasurface-based PTC made from a square lattice of multilayered spheres supporting qBICs, as shown in (A). (**C**) The band structure of the metasurface from (B) under static conditions, i.e., *m* = 0 in the spectral domain of interest. The gray dashed lines represent the light lines, and the red dotted line marks the spectral location of the flat band frequency $\omega_{\Gamma }$ at the Γ-point. (**D**) The normalized scattering cross section $\sigma_{\text{sca}}$ of the static isolated multilayered sphere used in (B) as a function of frequency. The sphere hosts a qBIC with a quality factor of $Q\approx 5\times 10^{4}$. (**E**) The band structure of the time-varying metasurface zoomed at the very narrow frequency range centered at $ℜ\left\{\omega \right\}=\omega_{m}/2$. An extremely low modulation amplitude $m=7\times 10^{-8}$ is used. (**F**) The further zoomed band structure in the highlighted region of (E) illustrates a finite momentum bandgap. (**G**) The corresponding imaginary part of the frequency inside the bandgap in (F) for a fixed $ℜ\left(\omega \right)/\omega_{m}=0.5$. The displayed modes have a symmetry compatible with that of *p*-polarized plane waves. (**H**) The imaginary part of the frequency inside the full bandgap for a fixed $ℜ\left(\omega \right)/\omega_{m}=0.5$. Here, $m=3.5\times 10^{-4}$ is used. The transparency of the bands in (C), (E), and (F) is directly proportional to the radiative losses that the underlying modes have. Moreover, $c_{0}$ is the speed of light in a vacuum.

To begin with, we analyze the BICs of static isolated multilayered spheres. We consider a sphere that consists of a core and two layers (see the inset in Fig. 2A). The core and the outer layer of the sphere are made from AZO. The dispersive permittivity of AZO is expressed using the Drude-Lorentz model (see section S1), where the corresponding parameters were taken from (*55*). The central layer is made from silica with a permittivity of 2.15. We denote the radii of the core, central, and outer layers of the sphere by $r_{0},r_{1},\text{and}\;r_{2}$, respectively. We assume $r_{0}=120\;\text{nm}$, $r_{1}=\eta r_{2}$, and $r_{2}=200\;\text{nm}$, where η is a variable. To demonstrate the existence of a true BIC, we first turn off the material losses in the AZO layers by setting the Drude-Lorentz loss parameter γ to 0. Next, we plot the normalized scattering cross section $\sigma_{\text{sca}}$ of the lossless sphere as a function of the frequency ω and the geometric parameter η (see Fig. 2A). The bright and dark regions in the color plot correspond to scattering zeros and resonances of the sphere, respectively. In the dashed box, the scattering zeros and the resonances approach each other, eventually merging at a point at $\eta =\eta_{\text{BIC}}\approx 0.6$. This coalescing of the zeros and the resonances is the signature for the existence of a true BIC at that point in parameter space (*54*). The BIC appears at the frequency where the permittivity of the AZO layers is zero (*54*). In the following, we study how this BIC affects the band structures of metasurfaces made from the multilayered spheres. Note that there exist other geometries, such as isolated core-shell spheres, that also support BICs, which are formed because of the presence of a vanishing-permittivity layer (*56*).

The considered metasurface is made from a square lattice of multilayered spheres (see Fig. 2B). The lattice period of the metasurface is $\Lambda =3r_{2}$. At first, we study the static case. We operate at $\eta =1.018\eta_{\text{BIC}}=0.611$ so that the spheres support a qBIC (see Fig. 2A). The band structure of the corresponding static metasurface is shown in Fig. 2C. We use the T-matrix method (based on the Mie theory) to compute the band structure (see Methods) (*52*, *57*). Here, we observe the emergence of flat bands due to the qBIC of the sphere. For comparison, we plot $\sigma_{\text{sca}}$ of the sphere as a function of the frequency ω in Fig. 2D. We note the existence of the qBIC of the sphere with the *Q*-factor ∼5 × 10^4^ at the frequency $\omega_{\text{qBIC}}$ in Fig. 2D, which explains the appearance of the flat bands in Fig. 2C. The shown qBIC has an electric dipolar character. Due to the spatial symmetry of the sphere, the qBIC resonance is threefold degenerate. Upon placing the sphere in the lattice, this degeneracy is lifted. Therefore, three distinct bands exist in Fig. 2C. The flat band frequency at the Γ-point is $\omega_{\Gamma }\approx \omega_{\text{qBIC}}$.

Next, we turn on the time modulation of the spheres. We assume that the background permittivity Δε of both AZO layers of the spheres is time periodic, i.e., $\text{Δε}\left(t\right)=\varepsilon_{\infty }\left[1+m(1+\text{cos}\left(\omega_{m}t\right)\right]$ (see sections S1 and S2). The silica layer remains static. We start by considering a very small value of *m*, i.e., $m=7\times 10^{-8}$. Note that, in addition to introducing time modulation, the pump wave offsets the background permittivity by an amount $m\varepsilon_{\infty }$. This offset leads to a small dc shift in the initial flat band frequency $\omega_{\Gamma }$. The dc-shifted flat band frequency is denoted by $\omega_{\Gamma }^{\prime }$. Next, we choose the modulation frequency $\omega_{m}$ such that $0.5\omega_{m}=\omega_{\Gamma }^{\prime }$. The numerical value of the modulation frequency is $\omega_{m}=2\omega_{\Gamma }^{\prime }=2\pi \times 459.4\;\text{THz}$. The band structure of the corresponding time-varying metasurface is shown in Fig. 2E. We observe that the band structure of the time-varying metasurface is formed by folding the band structure of the static metasurface about $ℜ\left(\omega \right)=0.5\omega_{m}$. In the time-varying case, the eigenmodes at $ℜ\left(\omega \right)=0.5\omega_{m}$ interact with each other. Choosing $0.5\omega_{m}$ at the flat band frequency $\omega_{\Gamma }^{\prime }$ enhances this interaction due to the small radiative losses of the eigenmodes of the flat band. Therefore, light interacts with the time-varying matter for a longer duration, leading to the emergence of a noticeable momentum bandgap at $0.5\omega_{m}$ even for vanishingly small *m*. We show the momentum bandgap in the magnified region around the Γ-point (Fig. 2F). Because the bandgap lies within the light cone, i.e., above the light lines, of the metasurface, we use the incident angle range Δθ corresponding to the $k_{∥}$ values that fall inside the bandgap to quantify the gap size. In particular, the shown gap corresponds to $\text{Δθ}=4^{\circ }$. This gap is a noticeable momentum bandgap since, even with *m* = 1, gaps of comparable size do not occur in effectively homogeneous PTCs (see section S4). Furthermore, we show the imaginary part of the frequency ℑ(ω) inside the momentum bandgap for a fixed $ℜ\left(\omega \right)/\omega_{m}=0.5$ in Fig. 2G. As expected, we observe that for the $k_{∥}$ values falling inside the gap, there exist modes with ℑ(ω)>0 responsible for wave amplification. Here, the plot is not symmetric about ℑ(ω)=0 due to the finite (although small) radiation loss in the metasurface.

Another interesting characteristic of the qBIC-assisted PTCs is that they can support full momentum bandgaps spanning over all $k_{∥}$ values. To open such a full bandgap, we increase the modulation amplitude to $m=3.5\times 10^{-4}$. The increased *m* leads to a slightly different flat band frequency $\omega_{\Gamma }^{\prime }$. Therefore, the modulation frequency is modified to $\omega_{m}=2\omega_{\Gamma }^{\prime }=2\pi \times 459.3\;\text{THz}$. All other parameters are kept unchanged. The corresponding imaginary part of the frequency ℑ(ω) inside the momentum bandgap for a fixed $ℜ\left(\omega \right)/\omega_{m}=0.5$ is shown in Fig. 2H. We find that there exist modes with ℑ(ω)>0 for all possible $k_{∥}$ values, leading to a full bandgap. In Fig. 2H, we show that ℑ(ω)>0 on the edges of the irreducible Brillouin zone (IBZ). However, here, as well as in the following examples with full bandgaps, we verified that ℑ(ω)>0 inside the entire IBZ. Note that our method enhances the $k_{∥}$ range for which the input waves can be amplified. However, similar to other PTC implementations, the frequency ω that gets amplified depends on the temporal boundary conditions. As discussed in (*40*), if the input wave (with its $k_{∥}$ inside the bandgap) exists in the PTC before the commencement of the time modulation, then it gets amplified regardless of its frequency ω. On the other hand, if the input wave enters the PTC after the modulation has commenced, then it gets amplified only if its frequency is $\omega =0.5\omega_{m}$.

Next, we show the effects of varying the *Q*-factor on the required modulation amplitude to maintain the bandgap. For a fair comparison, we always require a full bandgap. First, we vary the *Q*-factor by changing the geometry parameter η. We note that, to open a full bandgap using the time-varying metasurface, a threshold modulation amplitude $m=m_{\text{th}}$ is required.

**Fig. 3.**
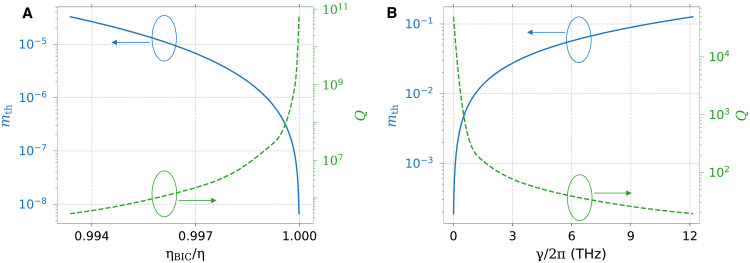
Variation of the threshold modulation amplitude and the *Q*-factor with system parameters. The threshold modulation amplitude $m_{\text{th}}$ of the multilayered sphere metasurface required to exhibit a full bandgap as a function of the geometry parameter $\eta =r_{1}/r_{2}$ [blue curve in (**A**)] and the Drude-Lorentz loss parameter γ [blue curve in (**B**)]. The *Q*-factor of the qBIC supported by the isolated static multilayered sphere as a function of η [green curve in (A)] and γ [green curve in (B)]. Note that $\eta /\eta_{\text{BIC}}=1.018$ is used in (B).

In Fig. 3A, we show $m_{\text{th}}$ as a function of η (blue curve). We find that as $\eta \to \eta_{\text{BIC}}$, $m_{\text{th}}\to 0$. This behavior is observed because, as $\eta \to \eta_{\text{BIC}}$, the *Q*-factor of the qBIC approaches infinity, i.e., Q→∞ (green curve in Fig. 3A). The high *Q*-factors reduce the radiation losses in the otherwise lossless system. Therefore, an arbitrarily small $m_{\text{th}}$ is sufficient to maintain a full bandgap.

So far, for a theoretical demonstration, we assumed the absence of material losses in the AZO layers. Next, we proceed to analyze the effect of material losses on $m_{\text{th}}$ to open a full bandgap. We plot $m_{\text{th}}$ as a function of the Drude-Lorentz loss parameter γ of the AZO layers in Fig. 3B (blue curve). Note that $\eta /\eta_{\text{BIC}}$ is fixed as $\eta /\eta_{\text{BIC}}=1.018$. We observe that upon increasing γ, the required $m_{\text{th}}$ increases. This behavior is expected because increasing the material losses decreases the *Q*-factor of the qBICs (green curve in Fig. 3B). Therefore, a higher $m_{\text{th}}$ is needed to open a full bandgap.

In particular, the experimentally reported value of γ in the spectral region of interest (the ENZ regime of AZO) is γ≈2π×12 THz (*55*). From Fig. 3B, we note that $m_{\text{th}}=0.13$ is required to open a full bandgap using this value of γ. The $m_{\text{th}}$ value corresponds to a pump intensity of 1.3 TW/cm^2^. In comparison, effectively homogeneous PTCs require $m_{\text{th}}∼1$ to even open finite bandgaps (see section S4).

It is worth mentioning that, while the qBICs of the multilayered spheres assist in decreasing $m_{\text{th}}$, assuming realistic losses, the corresponding metasurfaces still require rather high modulation amplitudes (and pump intensities) to open full bandgaps. In literature, metamaterial-based approaches have shown that the losses in the effective ENZ medium can be considerably reduced (*58*). Such an approach could be beneficial while using multilayered sphere qBICs to attain wide momentum bandgaps while maintaining low modulation amplitudes. Another challenge with multilayered sphere geometry is that it is difficult to fabricate. Keeping these difficulties in mind, we present a more practical design of the PTCs that uses the qBICs of dielectric cylinders as follows.

### PTCs based on metasurfaces made from germanium cylinders

Now, we begin with an isolated static germanium (Ge) cylinder supporting a high-*Q* qBIC. The material dispersion in the Ge cylinder is considered using the Drude-Lorentz model (see section S1). The Drude-Lorentz parameters of Ge are taken from (*59*). Realistic material losses in the Ge cylinder are included from the outset. The cylinder, with a radius of r=1030 nm and an aspect ratio of r/h≈0.703 (where *h* is the cylinder height), has been geometrically optimized following the strategy discussed in (*49*, *50*). This optimization leverages an avoided crossing between a Mie-type mode of azimuthal index *m* = 0 and principal (radial) index *n* = 1 and a neighboring m=0 Mie-type mode of different principal order (*n* = 2), where their interaction suppresses the radiative losses in the resulting hybrid mode.

Group theory analysis reveals that these m=0 modes belong to the $A_{2g}$ irreducible representation of the $D_{\infty h}$ point group that is relevant here. As a result, the hybrid mode is symmetry-protected and dark at axial incidence but emerges as a sharp qBIC under nonaxial *s*-polarized plane wave excitation (*60*). The qBIC is formed by the Friedrich-Wintgen mechanism (*49*). We highlight that this qBIC remains robust against the thermal expansion and thermo-optic effects for the considered pump intensities in the following (see section S5). Furthermore, because the qBIC has a symmetry compatible only with that of *s*-polarized plane waves, no resonance peak is observed for the *p*-polarized plane wave excitation.

**Fig. 4.**
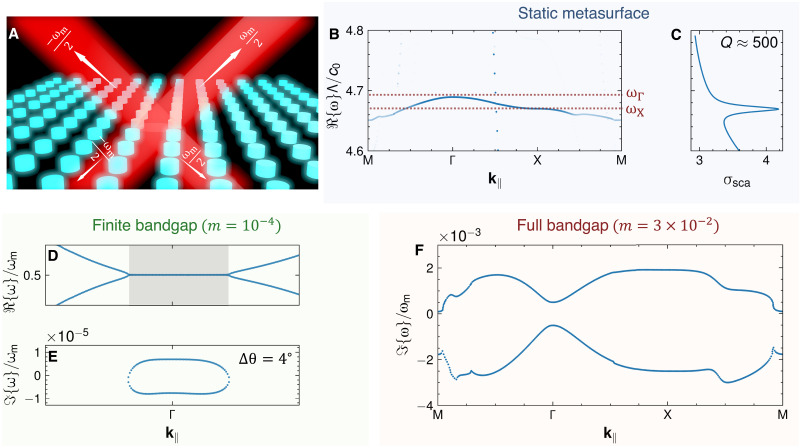
PTC based on cylinder metasurface. (**A**) A metasurface-based PTC made from a square lattice of resonant cylinders. (**B**) The band structure of the metasurface shown in (A) under static conditions, i.e., *m* = 0. Here, all the shown modes lie above the light lines. The red dotted lines mark the flat band frequencies $\omega_{\Gamma }$ and $\omega_{X}$ at the Γ- and X-points, respectively. (**C**) The rotationally averaged normalized scattering cross section $\sigma_{\text{sca}}$ of the isolated cylinder used in (A) as a function of frequency. The cylinder hosts a qBIC with the quality factor Q≈500. (**D**) The band structure of the time-varying metasurface zoomed at the very narrow frequency range centered at $ℜ\left\{\omega \right\}=\omega_{m}/2$ near the Γ-point, illustrating a finite bandgap. Here, $m=10^{-4}$ is used. (**E**) The corresponding imaginary part of the frequency inside the bandgap in (D) for a fixed $ℜ\left(\omega \right)/\omega_{m}=0.5$. The displayed mode branch has a symmetry compatible with that of *s*-polarized plane waves. (**F**) The imaginary part of the frequency inside the full bandgap for a fixed $ℜ\left(\omega \right)/\omega_{m}=0.5$. Here, $m=3\times 10^{-2}$ is used.

First, we analyze static metasurfaces made from such qBIC-supporting cylinders (see Fig. 4A). We assume the lattice period of the metasurface to be Λ=3.2r. The band structure of the metasurface under static conditions is shown in Fig. 4B, where we observe the emergence of a flat band. The flat band frequencies at the Γ- and X-points are $\omega_{\Gamma }$ and $\omega_{X}$, respectively. This flat band arises because of the existence of the aforementioned qBIC that the isolated static Ge cylinder hosts. For comparison, we show the rotationally averaged normalized scattering cross section $\sigma_{\text{sca}}$ of the cylinder as a function of frequency ω in Fig. 4C. In Fig. 4C, we observe the existence of the qBIC with Q≈500, which explains the existence of the flat band in Fig. 4B. Note that the *Q*-factor of the qBIC of the Ge cylinders is considerably lower than that of the multilayered spheres. This is because the spheres were assumed to be lossless. With realistic material losses, as here, finite dielectric nanoresonators cannot achieve arbitrarily high *Q*-factors (*50*). The *Q*-factor of the qBIC of a dielectric nanoresonator goes to infinity in the limit of vanishing material losses and for a diverging real part of the permittivity (*49*). However, as we demonstrate below, the *Q*-factor of the considered cylinders with realistic material parameters is sufficiently high to substantially reduce the required modulation amplitude for opening noticeable momentum bandgaps, compared to effectively homogeneous PTCs and dipolar Mie-resonant metasurfaces.

Next, we turn on the time modulation of the metasurface. We assume the background permittivity Δε of the Ge cylinders to be time periodic. The modulation amplitude is assumed to be $m=10^{-4}$. This value of *m* corresponds to a pump intensity of 0.7 GW/cm^2^. First, we aim at opening a finite bandgap near the Γ-point. Therefore, we choose the modulation frequency $\omega_{m}$ such that $0.5\omega_{m}=\omega_{\Gamma }^{\prime }$. Here, $\omega_{\Gamma }^{\prime }$ is the dc-shifted flat band frequency at the Γ-point. Numerically, $\omega_{m}=2\omega_{\Gamma }^{\prime }=2\pi \times 136\;\text{THz}$. We show the noticeable momentum bandgap hosted by the metasurface in the region around the Γ-point in Fig. 4D. We also plot the imaginary part of the frequency inside the bandgap in Fig. 4E. The displayed bandgap corresponds to an incident angle range of $\text{Δθ}=4^{\circ }$. We find that the cylinder-based metasurface requires an order of magnitude lower modulation amplitude than the recently reported dipolar Mie-resonant structure to achieve a bandgap of the same size (see section S6) (*43*). Moreover, even with *m* = 1, such a gap size is not attainable in effectively homogeneous PTCs (see section S4). The laser-induced damage threshold intensity of bulk Ge is 94 GW/cm^2^ using multishot pump pulses (*44*). Furthermore, the nanostructuring of Ge reduces its damage threshold by an order of magnitude, i.e., to a pump intensity of 9.4 GW/cm^2^ (*46*, *47*). Therefore, the modulation amplitude of $m=10^{-4}$ corresponding to the pump intensity of 0.7 GW/cm^2^ is well reachable in Ge using the optical Kerr effect without causing laser-induced damage to the underlying material. Note that, while Ge is almost transparent at $0.5\omega_{m}$, time modulation-induced upconverted frequencies may lie in its high material loss regime. Our T-matrix–based formalism accounts for such losses in all the examples studied in this work. At low modulation amplitudes, the coupling to those higher frequencies remains rather weak. Therefore, those material losses do not affect ℑ(ω) inside the bandgap appreciably.

Last, we show that, similar to the previous example, time-varying metasurfaces based on cylinders can also support full bandgaps. To illustrate this, we increase the modulation amplitude of the metasurface to $m=3\times 10^{-2}$. We choose the modulation frequency $\omega_{m}$ such that $0.5\omega_{m}=\omega_{X}^{\prime }$. Here, $\omega_{X}^{\prime }$ is the dc-shifted frequency of the eigenmode at the X-point. Our simulations show that this choice of $\omega_{m}$ requires the smallest modulation amplitude *m* to open a full bandgap as it maximizes the average overlap between the original modes and the band-folded modes induced by time modulation. Numerically, $\omega_{m}=2\omega_{X}^{\prime }=2\pi \times 134\;\text{THz}$. All other parameters are unchanged. We plot the imaginary part of the frequency inside the bandgap in Fig. 4F. We observe the existence of modes with ℑ(ω)>0 for all $k_{∥}$, leading to the demonstration of a full bandgap. The chosen value of *m* corresponds to a pump intensity of 210 GW/cm^2^. This pump intensity is about 20 times higher than the damage threshold of structured Ge under multishot laser pulses. However, using pump pulses with a reduced repetition rate can considerably increase the damage threshold (*45*). Therefore, a careful choice of the repetition rate may bring full bandgaps in reach in future works.

## DISCUSSION

In conclusion, we have shown that, by capitalizing on qBICs sustained in meta-atoms, we can substantially reduce the required modulation amplitude to observe noticeable momentum bandgaps in the corresponding metasurface-based PTCs. Using two different structures, we have shown the impact of material-induced as well as geometry-induced qBICs. Our theoretical analysis, based on metasurfaces composed of time-varying multilayered spheres, demonstrates that, by suppressing radiative losses in the meta-atoms, it is possible to achieve large momentum bandgaps for arbitrarily small modulation amplitudes, provided that absorption losses are negligible. Furthermore, we explored a viable route to realize a PTC based on metasurfaces that exploit qBICs supported by Ge cylinders. Using these metasurfaces, we demonstrate noticeable momentum bandgaps for modulation amplitudes as small as $m=10^{-4}$ corresponding to a pump intensity of 0.7 GW/cm^2^. This modulation amplitude is many orders of magnitude lower than that required in conventional homogeneous PTCs to open similarly-sized bandgaps. This value of *m* is achievable using the optical Kerr effect without causing laser-induced damage to the Ge cylinders. We anticipate that our results will be helpful in the experimental realization of PTCs at optical frequencies. We emphasize here that, apart from the discussed structures, the proposed framework can also be applied to other high-*Q* geometries to attain comparable results. These include structures with higher-order Mie resonances and those with more complicated meta-atom designs. In general, the threshold modulation amplitude $m_{\text{th}}$ required to open noticeable bandgaps decreases with increasing *Q*-factor. Moreover, we presented the application of the Mie theory to the time-varying metasurfaces made from multilayered spheres and finite-sized cylinders. We believe that our proposed numerical scheme will serve as a valuable tool for the further exploration of structured PTCs.

## METHODS

### Band structure calculation

We use the T-matrix method to evaluate the band structures of the time-varying metasurfaces (*57*, *61*). The T-matrix is closely linked to the well-known S-matrix used in scattering theory (see section S7) (*62*). According to the method, the incident field coefficients $A_{\text{inc}}$ and the scattered field coefficients $A_{\text{sca}}$, computed in a basis of vector spherical harmonics (VSHs), satisfy

$$A_{\text{sca}}={\left[\hat{I}-{\hat{T}}_{0}\left(\omega \right)\sum_{R\neq 0}{\hat{C}}^{\left(3\right)}\left(-R\right)e^{ik_{∥}\cdot R}\right]}^{-1}{\hat{T}}_{0}\left(\omega \right)A_{\text{inc}}$$ (1)

Here, $\hat{I}$ is the identity matrix, R is a lattice vector, and ${\hat{C}}^{\left(3\right)}$ is the translation matrix of VSHs (*57*, *63*, *64*). Furthermore, ${\hat{T}}_{0}$ is the T-matrix of an isolated time-varying scatterer. The T-matrices of isolated time-varying multilayered spheres can be evaluated using the Floquet-Mie theory, while those of isolated time-varying cylinders can be computed using the dynamic extended boundary condition method (EBCM) (*65*–*67*). Moreover, the lattice summation shown in Eq. 1 is calculated using the treams package (*64*). To allow evaluating the lattice sums in the lower half of the complex frequency plane [i.e., for ℑ(ω)<0], the branch cut of the incomplete gamma function is rotated down from the typical negative real axis by π/2. The incomplete gamma function with shifted branch cut is evaluated via its relation to the unshifted incomplete gamma function [see equation (8.2.9) of (*68*)]. Here, we use *J* as the total number of frequencies that the time-varying structure scatters upon a monochromatic incidence to construct ${\hat{C}}^{\left(3\right)}$ and ${\hat{T}}_{0}$. Furthermore, these matrices consist of terms up to the maximum multipolar order $l_{\text{max}}$. In particular, for computing the T-matrix of cylinders, using the dynamic EBCM, we use the maximum multipolar order $l_{\text{cut}}$. Here, $l_{\text{cut}}>l_{\text{max}}$ is chosen to ensure sufficient numerical convergence. However, after computing the T-matrix, we retain its terms only up to the order $l_{\text{max}}$. Therefore, the dimension of the matrices ${\hat{C}}^{\left(3\right)}$ and ${\hat{T}}_{0}$ is $2{\mathrm{Jl}}_{\text{max}}\left(l_{\text{max}}+2\right)$. We provide the used *J*, $l_{\text{max}}$, and $l_{\text{cut}}$ values for generating the results in this article in section S8.

For calculating the spectral location of an eigenmode, we require $A_{\text{sca}}\neq 0$ while $A_{\text{inc}}=0$ (*69*, *70*). Using Eq. 1, we find that this requirement is satisfied if the following holds

$$\underset{|\Psi \left(k_{∥},\omega \right)|}{\underbrace{|\hat{I}-{\hat{T}}_{0}\left(\omega \right)\sum_{R\neq 0}{\hat{C}}^{\left(3\right)}\left(-R\right)e^{ik_{∥}\cdot R}|}}=0$$ (2)

Therefore, those ω and $k_{∥}$ values that satisfy Eq. 2 characterize the spectral location of an eigenmode in the band structure of the metasurface. The band structure is plotted as a scatter plot of the spectral locations of the eigenmodes of the metasurface. Due to radiative loss and the gain/loss introduced by the time modulation, the bands generally have complex-valued ω. Locating the resonance frequencies of eigenmodes is a well-known computational problem for static photonic resonators. Recently, the adaptive Antoulas-Anderson (AAA) algorithm (*71*) has been introduced to the field of resonant nanophotonics (*72*) to locate the eigenfrequencies as poles of some optical response function. The AAA algorithm was shown to be well-suited for locating the resonances of coupled clusters with a finite number of meta-atoms, leveraging the T-matrix method (*73*). Here, we generalize this approach to periodic lattices of time-varying meta-atoms. The AAA algorithm is used to find a good rational approximation $r_{k_{∥}}\left(\omega \right)$ given by

$$r_{k_{∥}}\left(\omega \right)\approx f\left(k_{∥},\omega \right)=\frac{1}{|\Psi \left(k_{∥},\omega \right)|}$$

based on a sufficient number of sample evaluations of $|\Psi \left(k_{∥},\omega \right)|$ for a fixed $k_{∥}$. Then, performing a pole search of the function $r_{k_{∥}}\left(\omega \right)$ allows us to find the ω and $k_{∥}$ values that satisfy Eq. 2.

## Supplementary Materials

### This PDF file includes:

Supplementary Text Figs. S1 to S6 References
