# Supplementary material for: Photonic time crystals assisted by quasi-bound states in the continuum
Source: Sci Adv. 2026 Aug 12;12(33):eaed4055. doi: 10.1126/sciadv.aed4055 (PMC13464636; doi:10.1126/sciadv.aed4055)
Supplement: Supplementary file 1 [file sciadv.aed4055_sm.pdf]

Science Advances
AAAS

Supplementary Materials for

Photonic time crystals assisted by quasi-bound states in the continuum

Puneet Garg *et al.*

Corresponding author: Puneet Garg, puneet.garg@kit.edu; Viktar Asadchy, viktar.asadchy@aalto.fi;
Carsten Rockstuhl, carsten.rockstuhl@kit.edu

Sci. Adv. **12**, eaed4055 (2026)
DOI: 10.1126/sciadv.aed4055

This PDF file includes:

Supplementary Text
Figs. S1 to S6
References

Supplementary Text

S1 Time modulation due to the optical Kerr effect

We use the optical Kerr effect as the source of time modulation in the discussed metasurface scenarios. In a homogeneous nonlinear Kerr medium, the physical (real-valued) polarization density for the signal field can be written as (*40, 53, 74*)

$$\mathbf{P}(\mathbf{r},t) = \varepsilon_0 \int_{-\infty}^{\infty} R^{(1)}(t-\tau)\,\mathbf{E}(\mathbf{r},\tau)\mathrm{d}\tau + \varepsilon_0 \chi^{(3)} E_{\mathrm{pump}}^2(\mathbf{r},t)\,\mathbf{E}(\mathbf{r},t)\,, \tag{S1}$$

where $\mathbf{E}$ and E_{pump} are the real-valued signal and pump electric fields, respectively. We use the undepleted pump approximation. Moreover, the polarization of the pump field is also fixed. Here, $R^{(1)}(t-\tau)$ is the dispersive linear response function, and $\chi^{(3)}$ is the third-order nonlinear susceptibility of the medium. Note that the third-order nonlinear response function of the medium is defined as $R^{(3)}(t-\tau) = \delta(t-\tau)\chi^{(3)}$, where the Dirac delta distribution, $\delta(t-\tau)$, represents the instantaneous nature of the third-order nonlinear response of the medium to the electric fields (*74*). Physically, this instantaneous response is facilitated by the virtual excitation and relaxation of the electrons in the nonlinear medium (*74*). Note that the polarization density model in Eq. (S1) to describe the physics of PTCs has been proposed in (*40, 53, 74*). Next, we use a pump field given by $E_{\mathrm{pump}} = E_{\mathrm{p}}(\mathbf{r})\cos(\omega_{\mathrm{p}} t + \phi(\mathbf{r}))$. Therefore, Eq. (S1) can be written as

$$\mathbf{P}(\mathbf{r},t) = \varepsilon_0 \int_{-\infty}^{\infty} R^{(1)}(t-\tau)\,\mathbf{E}(\mathbf{r},\tau)\mathrm{d}\tau + \varepsilon_0 \chi^{(3)} E_{\mathrm{p}}^2(\mathbf{r})\cos^2(\omega_{\mathrm{p}} t + \phi(\mathbf{r}))\,\mathbf{E}(\mathbf{r},t) \tag{S2}$$

$$= \varepsilon_0 \int_{-\infty}^{\infty} R^{(1)}(t-\tau)\,\mathbf{E}(\mathbf{r},\tau)\mathrm{d}\tau + \varepsilon_0 \frac{\chi^{(3)}}{2} E_{\mathrm{p}}^2(\mathbf{r})\left[1 + \cos(2\omega_{\mathrm{p}} t + 2\phi(\mathbf{r}))\right]\mathbf{E}(\mathbf{r},t)\,. \tag{S3}$$

For simplicity, we assume that the pump field is independent of the spatial coordinate $\mathbf{r}$. We provide a justification for this homogeneous pump approximation in Supplementary Section S2. Therefore, we write

$$\mathbf{P}(\mathbf{r},t) = \varepsilon_0 \int_{-\infty}^{\infty} \left\{ R^{(1)}(t-\tau) + \delta(t-\tau)\frac{\chi^{(3)}}{2} E_{\mathrm{p}}^2 \left[1 + \cos(2\omega_{\mathrm{p}} t + 2\phi)\right] \right\} \mathbf{E}(\mathbf{r},\tau)\mathrm{d}\tau\,. \tag{S4}$$

The optical Kerr effect gives rise to the following time-dependent effective linear response function

$$R^{(1)}_{\text{eff}}(t, t-\tau) = R^{(1)}(t-\tau) + \delta(t-\tau)\frac{\chi^{(3)}}{2}E_{\text{p}}^2\left[1+\cos(2\omega_{\text{p}}t+2\phi)\right] . \quad \text{(S5)}$$

We rewrite $R^{(1)}(t-\tau) = \chi_\infty\delta(t-\tau) + R^{(1)}_{\text{disp}}(t-\tau)$, where $R^{(1)}_{\text{disp}}$ is the dispersive part of the linear response function and χ_∞ is the non-dispersive background susceptibility of the underlying static medium. Therefore

$$R^{(1)}_{\text{eff}}(t, t-\tau) = R^{(1)}_{\text{disp}}(t-\tau) + \delta(t-\tau)\Delta\chi(t) , \quad \text{(S6)}$$

where

$$\Delta\chi(t) = \chi_\infty + \frac{\chi^{(3)}E_{\text{p}}^2}{2}\left[1+\cos(2\omega_{\text{p}}t+2\phi)\right] . \quad \text{(S7)}$$

Moreover, we can write

$$\Delta\varepsilon(t) = 1 + \Delta\chi(t) = \varepsilon_\infty\left\{1 + \frac{\chi^{(3)}E_{\text{p}}^2}{2\varepsilon_\infty}\left[1+\cos(2\omega_{\text{p}}t+2\phi)\right]\right\} . \quad \text{(S8)}$$

Here, $\Delta\chi(t)$ and $\Delta\varepsilon(t)$ denote the background susceptibility and permittivity of the time-varying medium, respectively. In addition, χ_∞ and ε_∞ $(= 1+\chi_\infty)$ represent the corresponding parameters of the underlying static medium. Note that this time-varying background permittivity model has been employed to explain the experimental results in (*10*). Next, we define the modulation frequency $\omega_{\text{m}} = 2\omega_{\text{p}}$, and modulation amplitude $m = \frac{\chi^{(3)}E_{\text{p}}^2}{2\varepsilon_\infty}$. Hence

$$\Delta\varepsilon(t) = \varepsilon_\infty\left\{1 + m\left[1+\cos(\omega_{\text{m}}t)\right]\right\} . \quad \text{(S9)}$$

Here, the initial phase ϕ of the pump field is set to zero without loss of generality, as any nonzero phase merely amounts to the time-translation of $\Delta\varepsilon(t)$ by $\Delta t = 2\phi/\omega_{\text{m}}$. Such a time-translation does not affect the band structures of the PTCs. This phase insensitivity of PTCs has been discussed in detail in (*32*).

Next, we perform the Fourier transform of Eq. (S5) to obtain (*66*)

$$\begin{aligned}\tilde{R}^{(1)}_{\text{eff}}(\omega-\omega', \omega') = {} & \delta(\omega-\omega')\sum_j \frac{\omega_{\text{p}j}^2}{\omega_{0j}^2 - i\gamma_j - \omega^2} \\ & + \chi_\infty\left\{\delta(\omega-\omega') + \frac{\chi^{(3)}E_{\text{p}}^2}{2\chi_\infty}\left[\delta(\omega-\omega') + \frac{1}{2}\Big(\delta(\omega-\omega'-\omega_{\text{m}}) + \delta(\omega-\omega'+\omega_{\text{m}})\Big)\right]\right\} .\end{aligned} \quad \text{(S10)}$$

Here, $\tilde{R}^{(1)}_{\text{eff}}(\omega - \omega', \omega')$ is the Fourier transform of the linear response function $R^{(1)}_{\text{eff}}(t, t - \tau)$. Moreover, we use the Drude-Lorentz model to consider material dispersion, with $\omega_{\text{p}j}$ being the plasma frequency, ω_{0j} the resonance frequency, and γ_j the loss parameter of the jth Drude-Lorentz oscillator. Note that in the main text, we use the nomenclature $\gamma_1 = \gamma$. We have used the Drude-Lorentz parameters of Ge from (*59*) and $\chi^{(3)}$ from (*41*). For AZO, the corresponding parameters are taken from (*55*).

S2 Homogeneous pump approximation

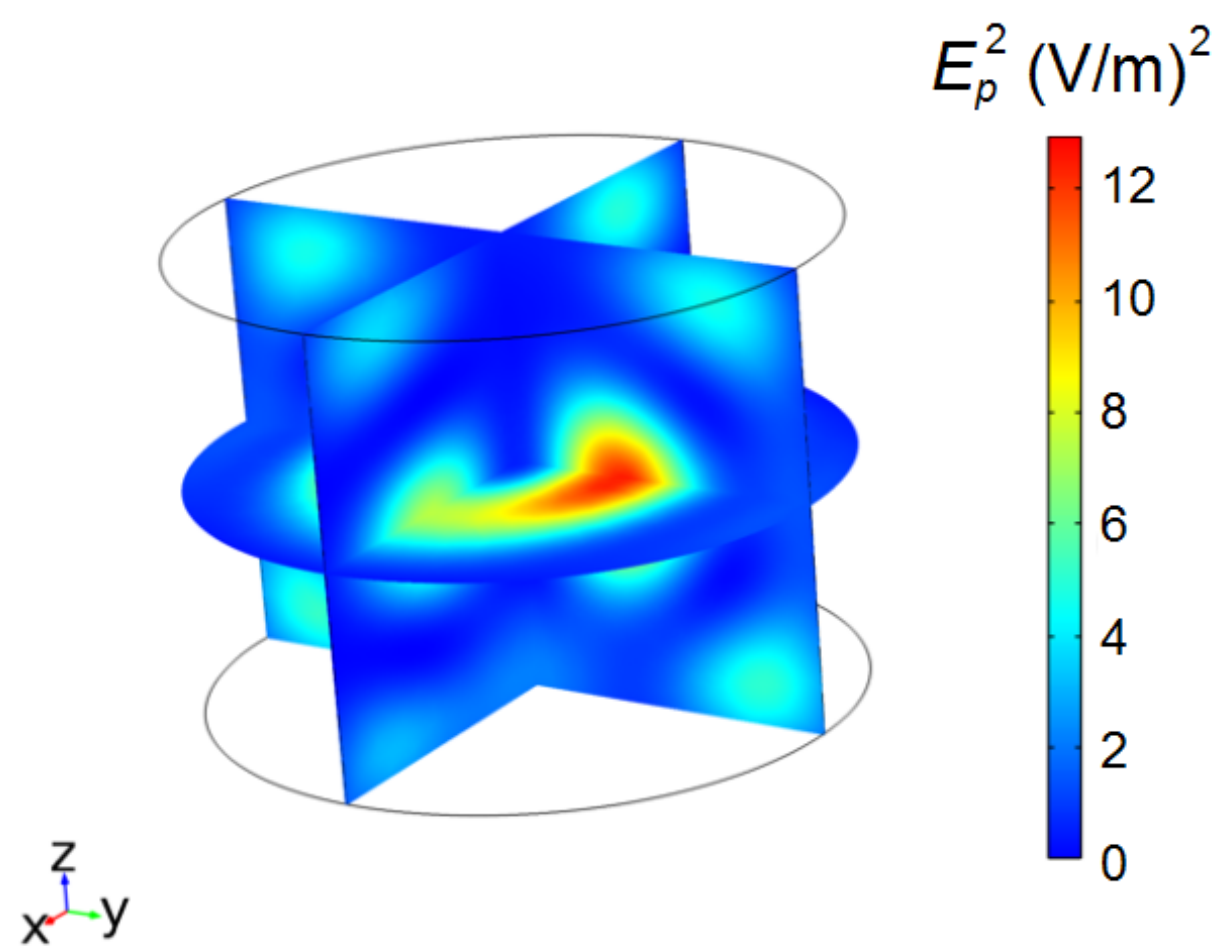

Figure S1: **Pump-induced spatial inhomogeneity of modulation amplitude**. Spatial distribution of the pump-induced electric field (squared amplitude), *i.e.*, E_{p}^2 inside a single cylindrical germanium nanoresonator of the metasurface at the pump frequency $\omega_{\text{p}} = 0.5\omega_{\text{m}}$. Here, $0.5\omega_{\text{m}}$ is chosen to be the same as that in Figs. 4(D)–(E). The pump field is a *p*-polarized plane wave of amplitude 1 V/m, incident at 5° with respect to the metasurface normal, a configuration that does not excite the high-Q modes of the flat band. Although the internal field exhibits spatial inhomogeneity, the Kerr-induced background permittivity modulation remains perturbative and can be effectively represented by a homogeneous pump amplitude in the full-wave calculations.

The Kerr-induced temporal modulation experienced by the signal wave is governed by the pump field

distribution inside the nanoresonators. In general, the internal pump field, and thus the induced background permittivity change, are spatially inhomogeneous. However, since the Kerr-induced background permittivity modulation remains perturbative with respect to the static background permittivity, its detailed spatial structure influences the signal dynamics only through overlap integrals between the space-dependent modulation profile $m(\mathbf{r})$ and the relevant eigenmodes of the underlying *static* scattering structure. In particular, the coupling coefficient of the original mode and the time modulation-induced mode on the flat band can be written as (*75*)

$$\kappa = \int_V \mathbf{E}^*_{\mathrm{res}}(\mathbf{r})\, m(\mathbf{r})\, \mathbf{E}_{\mathrm{res}}(\mathbf{r})\mathrm{d}V\,, \tag{S11}$$

where $\mathbf{E}_{\mathrm{res}}(\mathbf{r})$ and $\mathbf{E}^*_{\mathrm{res}}(\mathbf{r})$ are the spatial field profiles of the eigenmodes on the flat bands corresponding to the frequencies $0.5\omega_{\mathrm{m}}$ and $-0.5\omega_{\mathrm{m}}$, respectively, and V is the volume of the nanoresonator inside the lattice. Note that $\mathbf{E}_{\mathrm{res}}(\mathbf{r})$ and $\mathbf{E}^*_{\mathrm{res}}(\mathbf{r})$ correspond to the field profiles of the high-Q modes of the underlying *static* metasurface. They can be excited by the signal wave. Moreover, the pump wave couples these modes through a time modulation characterized by the space-dependent modulation amplitude profile $m(\mathbf{r})$. The formulation defined by Eq. (S11) has been derived and numerically verified in (*75*). Next, Eq. (S11) can be approximated to define an effective modulation amplitude as

$$\kappa = \int_V \mathbf{E}^*_{\mathrm{res}}(\mathbf{r})\, m(\mathbf{r})\, \mathbf{E}_{\mathrm{res}}(\mathbf{r})\mathrm{d}V \approx \int_V \mathbf{E}^*_{\mathrm{res}}(\mathbf{r})\, m_{\mathrm{eff}}\, \mathbf{E}_{\mathrm{res}}(\mathbf{r})\mathrm{d}V\,, \tag{S12}$$

so the spatial structure of the modulation does not enter explicitly. Accordingly, the spatially inhomogeneous modulation can be equivalently represented by a constant effective amplitude m_{eff}. In particular, when the pump is tuned to the frequency of the flat band, the excitation of the high-Q resonance by the pump can be avoided by an appropriate choice of the pump incidence direction and polarization, thereby avoiding resonant field localization inside the nanoresonators that would otherwise lead to strong spatial inhomogeneities in the induced background permittivity modulation.

To be more specific, we study the Kerr effect for the finite bandgap case presented in Fig. 2. The relevant quantity for the Kerr effect is the squared field amplitude $E_{\mathrm{p}}^2(\mathbf{r})$ of the pump wave. The frequency ω_{p} of the pump wave is identical to $0.5\omega_{\mathrm{m}}$ used in Figs. 4(D)–(E). Moreover, the structural and material parameters of the metasurface are also chosen to be the same as those used

in Figs. 4(D)–(E). We assume p-polarized light incident at an angle 5° with respect to the axis normal to the metasurface. We point out that, for this polarization, the high-Q modes of the flat band in Fig. 2(B) cannot be excited. The resulting $E_{\mathrm{p}}^2(\mathbf{r})$ profile inside a cylindrical nanoresonator of the metasurface is shown in Fig. S1. Here, we observe that the pump field distribution inside the nanoresonator is spatially inhomogeneous; however, thanks to the careful choice of the incident polarization, the high-Q mode is not excited. Furthermore, because the modulation amplitude m remains perturbative, the approximation in Eq. (S12) can be reliably applied.

S3 Comparison of the meta-atom and lattice qBICs

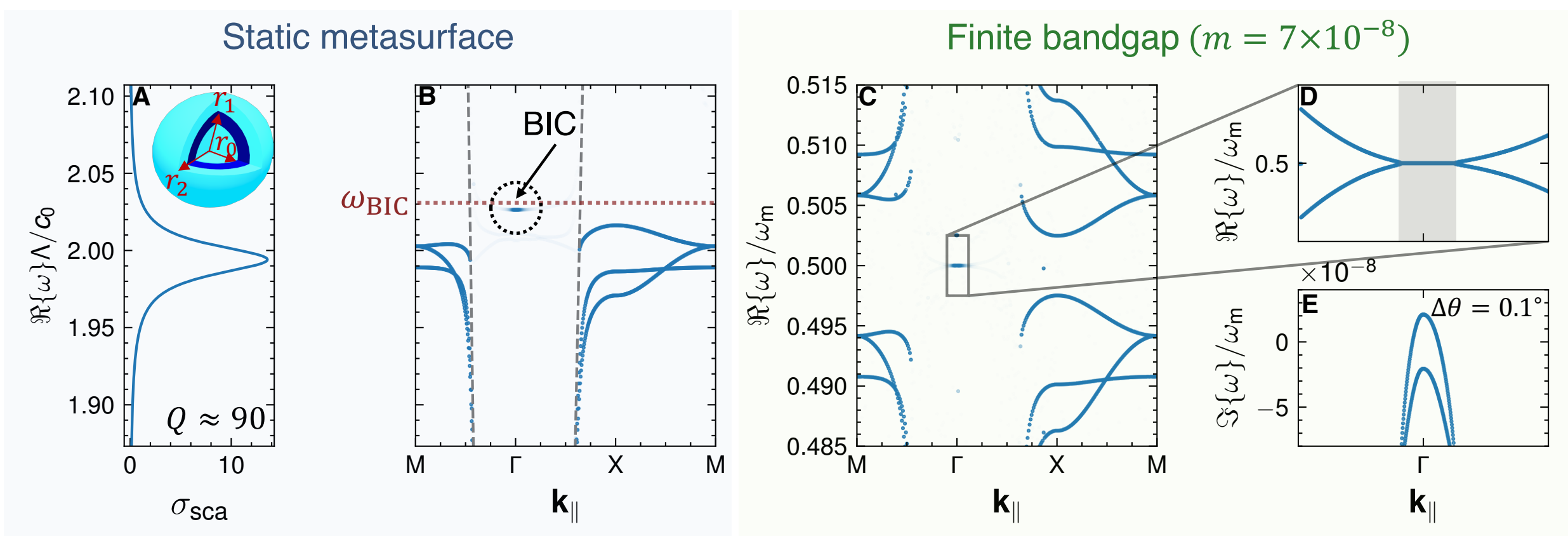

Figure S2: **PTC assisted by lattice-induced qBIC**. (**A**) The normalized scattering cross section σ_{sca} of the static multilayered sphere (see inset) as a function of the frequency ω. The sphere consists of a core and two layers. The core and the outer layer of the sphere are made from AZO. The middle layer is made from silica. Note that material losses are neglected here to clearly illustrate the effects. (**B**) The band structure of the metasurface formed by a square lattice of multilayered spheres under static conditions, *i.e.*, $m = 0$ in the spectral domain of interest. The red-dotted line marks the spectral location of the true lattice BIC at the Γ-point. (**C**) The band structure of the time-varying metasurface. An extremely low modulation amplitude $m = 7\times10^{-8}$ is used. (**D**) The further zoomed band structure in the highlighted region of (C) illustrates a finite momentum bandgap. The displayed mode has a symmetry compatible with that of p-polarized plane waves. (**E**) The corresponding imaginary part of the frequency inside the bandgap in (D) for a fixed $\Re(\omega)/\omega_{\mathrm{m}} = 0.5$.

In this section, we show the influence of the qBICs of the metasurfaces formed by lattice interactions on momentum bandgap sizes. We also compare the results obtained thanks to these lattice qBICs with those of the meta-atom qBICs used in the main text. We consider a metasurface made from a square lattice of lossless multilayered spheres (see the inset of Fig. S2(A)). The core and the outer layer of the spheres are made from lossless AZO. The middle layer is made from lossless silica. To clearly illustrate the impact of the lattice qBICs, the isolated multilayered sphere is designed such that it sustains a relatively low-Q resonance. We use, $r_2 = 200$ nm, $r_0 = 66.67$ nm, and $\eta = 0.9$.

The normalized scattering cross section σ_{sca} of the static multilayered sphere is shown in Fig. S2(A). Here, we observe that the multilayered sphere hosts an electric dipolar Mie resonance with $Q \approx 90$. This Q-factor is three orders of magnitude lower than that of the qBIC resonance used in Fig. 2(D). Next, we plot the band structure of the corresponding static metasurface in Fig. S2(B). The metasurface is formed by a square lattice of multilayered spheres with lattice period $\Lambda = 3r_2$. We observe the existence of flat bands in Fig. S2(B). However, due to the relatively low Q-factors of the multilayered spheres, the bands are considerably less flat than those shown in Fig. 2(C). This metasurface also supports a true symmetry-protected lattice BIC at the frequency ω_{BIC} at the Γ-point. Such a BIC is formed due to the lattice interactions in the metasurface (*76*). We note that, despite the presence of a lattice BIC, the flatness of the bands of the metasurface is controlled by the Q-factors of the individual meta-atoms.

To study the impact of the lattice qBICs on the sizes of the momentum bandgaps, we turn on the time modulation of the meta-atoms. We use $m = 7 \times 10^{-8}$, which is identical to that used in Fig. 2(E). Moreover, we choose $0.5\omega_{\mathrm{m}} \approx \omega'_{\mathrm{BIC}}$. Here, ω'_{BIC} is the DC-shifted frequency of the lattice BIC. Numerically, $\omega_{\mathrm{m}} = 2\pi \times 161$ THz. The band structure of the corresponding time-varying metasurface is shown in Fig. S2(C). Here, we note that due to the lower flatness of the bands, upon time modulation, a smaller number of modes interact with each other as compared to those in Fig. 2(E) (see the gray box). Therefore, a relatively smaller gap opens despite the same modulation amplitude m. We show the corresponding momentum bandgap in Fig. S2(D). The imaginary part of the frequency $\Im(\omega)$ inside the bandgap is shown in Fig. S2(E). Here, the gap corresponds to $\Delta\theta = 0.1°$. This gap size is 40 times lower than that shown in Figs. 2(F)–(G).

Importantly, the lattice qBICs of the metasurfaces allow for the existence of narrow momentum bandgaps with extremely low m. However, it is the qBICs of the individual meta-atoms that assist in

expanding the momentum bandgap sizes while simultaneously lowering the modulation amplitude requirements. Physically, the strong localization of the fields inside the individual meta-atoms reduces the spatial dispersion in the lattice while simultaneously allowing for a large interaction time of light with time-varying matter.

S4 Comparison with effectively homogeneous media

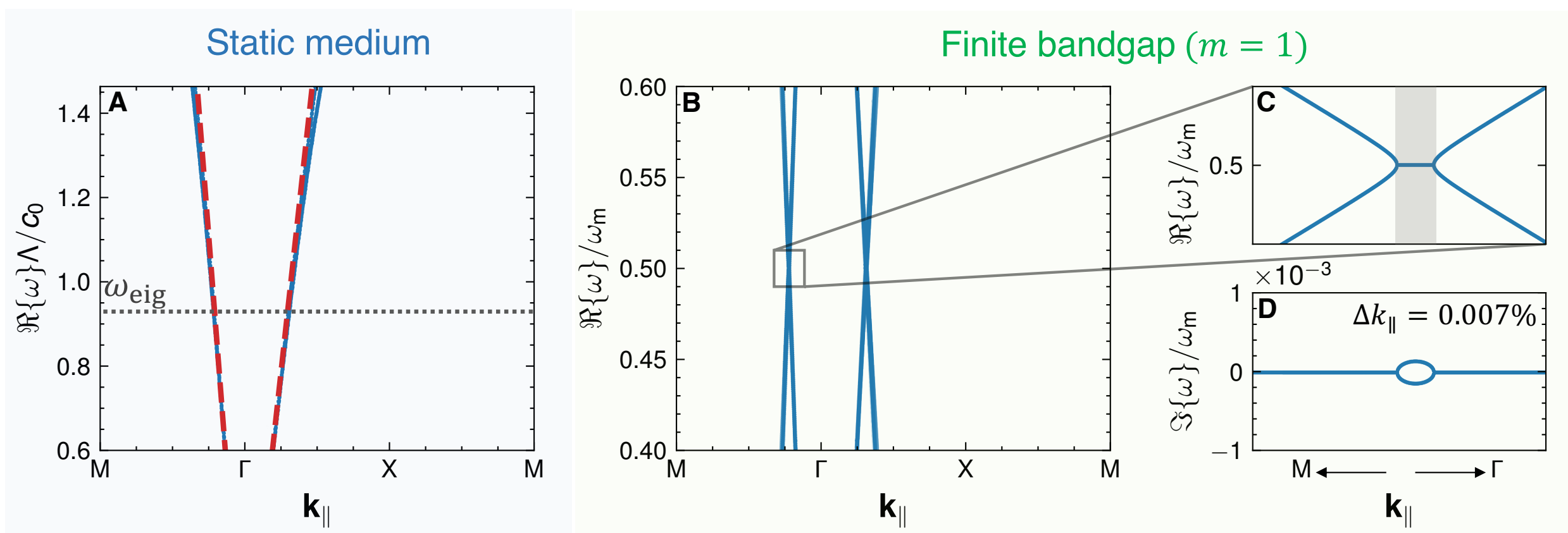

Figure S3: **Effectively homogeneous medium build based on the metasurface of multilayered spheres**. (**A**) The band structure of the effectively homogeneous medium under static conditions, *i*.e., $m = 0$. The homogeneous medium is numerically modeled by operating the metasurface in Fig. 2 in its spatially homogenizable regime, *i.e.*, $\Re(\omega)\Lambda/c_0 < \pi$. The gray-dotted line marks the spectral location of an eigenmode in the M-Γ region. Moreover, the red-dashed lines represent the light lines. (**B**) The band structure of the medium in the time-varying case. A high modulation amplitude $m = 1$ is used. (**C**) The further zoomed band structure in the highlighted region of (B) illustrates a finite momentum bandgap. The displayed mode has a symmetry compatible with that of p-polarized plane waves. (**D**) The corresponding imaginary part of the frequency inside the bandgap in (C) for a fixed $\Re(\omega)/\omega_{\text{m}} = 0.5$.

In the main text, we showed that by harnessing the flat bands arising due to the qBICs in metasurface-based PTCs, noticeable momentum bandgaps are reached while requiring considerably low modulation amplitudes. Here, we show that in effectively homogeneous media, despite much higher modulation amplitudes, the gap sizes remain rather small.

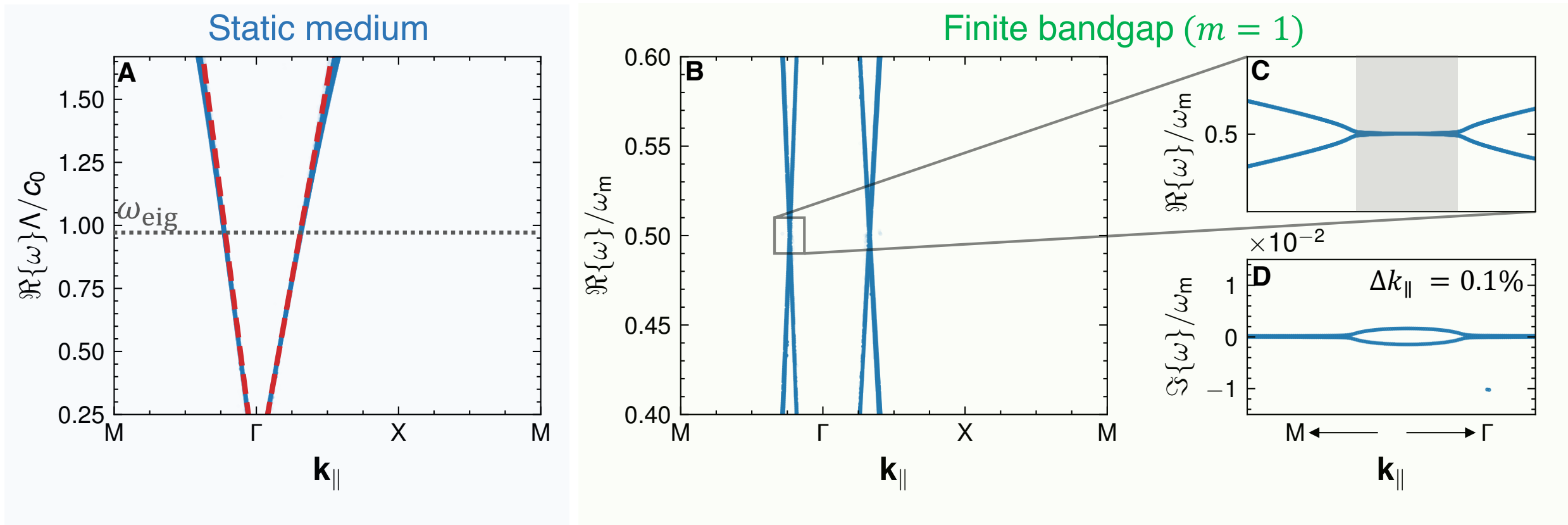

Figure S4: **Effectively homogeneous medium build based on the metasurface of cylinders**. (**A**) The band structure of the effectively homogeneous medium under static conditions, *i*.e., $m = 0$. The homogeneous medium is numerically modeled by operating the metasurface in Fig. 4 in its spatially homogenizable regime, *i.e.*, $\Re(\omega)\Lambda/c_0 < \pi$. The gray-dotted line marks the spectral location of an eigenmode in the M-Γ region. Moreover, the red-dashed lines represent the light lines. (**B**) The band structure of the medium in the time-varying case. A high modulation amplitude $m = 1$ is used. (**C**) The further zoomed band structure in the highlighted region of (B) illustrates a finite momentum bandgap. The displayed mode has a symmetry compatible with that of *s*-polarized plane waves. (**D**) The corresponding imaginary part of the frequency inside the bandgap in (C) for a fixed $\Re(\omega)/\omega_{\rm m} = 0.5$.

To numerically model homogeneous media, we operate the metasurfaces used in the main text in their spatially homogenizable regime. A metasurface is homogenizable if the wavelength λ of the considered eigenmode is at least twice its lattice period Λ, *i.e.*, $\lambda > 2\Lambda$. In terms of the eigenmode frequency ω, this condition can be written as $\Re(\omega)\Lambda/c_0 < \pi$.

First, we consider the effectively homogeneous medium modeled by operating the metasurface used in Fig. 2 in its spatially homogenizable regime. We choose the same system parameters as those used to plot Fig. 2(C) in the simulations. In particular, the multilayered spheres are assumed to be lossless. The band structure of the static medium in the homogenizable regime is shown in Fig. S3(A). We observe the characteristic linear dispersion of the homogeneous media. We note that the shown bands appear below the light lines. Here, $\omega_{\rm eig}$ is the frequency of the eigenmode in the M-Γ region. Next, we choose the modulation frequency $\omega_{\rm m}$ such that the linear bands interact

upon time modulation. In particular, $0.5\omega_{\mathrm{m}} = \omega'_{\mathrm{eig}}$. Here, ω'_{eig} is the DC-shifted frequency of the eigenmode in the M-Γ region. Numerically, $\omega_{\mathrm{m}} = 2\pi \times 151$ THz. Furthermore, we use a high modulation amplitude of $m = 1$. The band structure in the time-varying case is shown in Fig. S3(B). Here, we observe the crossing of the bands. Due to the linearity of the bands, upon time modulation, very few modes interact with each other as compared to those in Fig. 2(E) (see the gray box). Therefore, an extremely small band gap opens despite the large modulation amplitude. We show the corresponding momentum bandgap in Fig. S3(C). The imaginary part of the frequency $\Im(\omega)$ inside the bandgap is shown in Fig. S3(D). To quantify the gap size, we cannot use the angular bandwidth as done in the main text, since the bandgap of the effectively homogeneous medium appears below the light line. Therefore, we define the new gap size metric $\Delta k_{\|}$ as

$$\Delta k_{\|} = \frac{\delta k_{\|}^{\mathrm{M}} + \delta k_{\|}^{\mathrm{X}}}{L} \,. \tag{S13}$$

Here, $\delta k_{\|}^{\mathrm{M}}$ and $\delta k_{\|}^{\mathrm{X}}$ are the momentum extents of the gap in the M-Γ, and Γ-X regions of the IBZ, respectively. Moreover, L is the total momentum extent of the M-Γ-X region, which equals $(\sqrt{2} + 1)\frac{\pi}{a}$.

Using the gap size metric in Eq. (S13), we find that the bandgap size in Figs. S3(C)–(D) is $\Delta k_{\|} = 0.007\%$ for $m = 1$. On the other hand, the gap size in Figs. 2(F)–(G) is $\Delta k_{\|} = 3\%$ for $m = 7 \times 10^{-8}$. Therefore, we conclude that homogeneous media support a much smaller bandgap, even for huge modulation amplitudes, than the corresponding qBIC-assisted PTCs.

Next, we tune the cylinder-based metasurface shown in Fig. 4 to its spatially homogenizable regime. Here, we choose the same system parameters as those used to plot Fig. 4(B) in the simulations. The material losses in Ge cylinders are fully considered. The band structure of the medium in the static case is shown in Fig. S4(A). We again observe characteristic linear dispersion of homogeneous media. Here, ω_{eig} is the frequency of the eigenmode in the M-Γ region. The modulation frequency is again chosen such that the linear bands cross upon time modulation. In particular, $0.5\omega_{\mathrm{m}} = \omega'_{\mathrm{eig}}$, where ω'_{eig} is the DC-shifted frequency of the eigenmode in the M-Γ region. Numerically, $\omega_{\mathrm{m}} = 2\pi \times 29$ THz. Furthermore, a large modulation amplitude of $m = 1$ is chosen. The band structure in the time-varying case is shown in Fig. S4(B). Moreover, the zoomed band structure in the region of band crossing, and the corresponding imaginary part of the frequency $\Im(\omega)$ inside the bandgap is shown in Figs. S3(C) and (D), respectively. We observe that

for $m = 1$, a gap of size $\Delta k_{\parallel} = 0.1\%$ is observed. On the other hand, the gap size in Figs. 4(D)–(E) is $\Delta k_{\parallel} = 4\%$ for $m = 10^{-4}$. Therefore, we find that homogeneous media support approximately 40 times smaller bandgap even for 4 orders of magnitude higher modulation amplitudes than the corresponding qBIC-assisted PTCs.

S5 qBICs of germanium cylinders

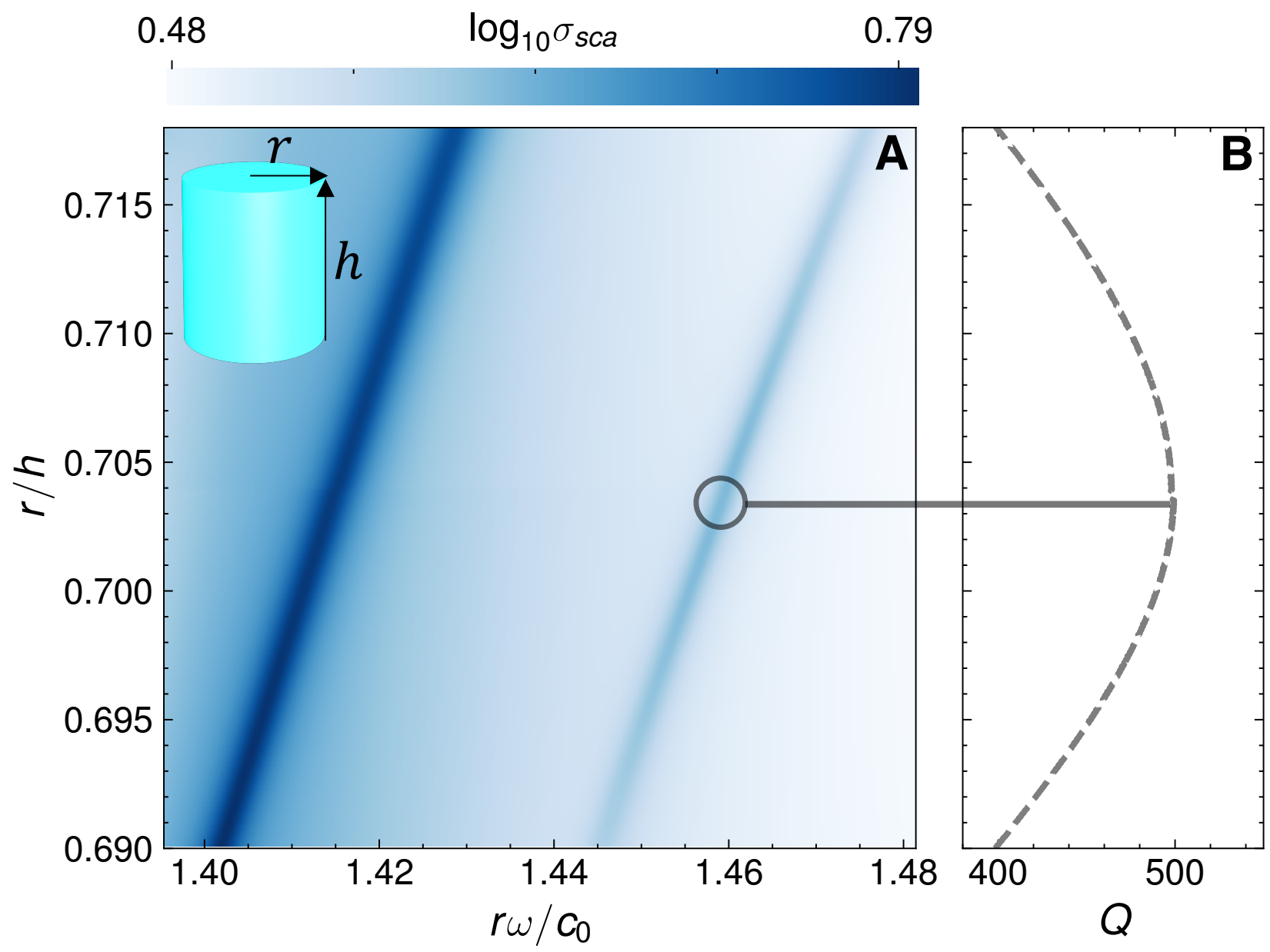

Figure S5: **The qBIC of the isolated static cylinder**. (**A**) The rotationally averaged normalized scattering cross section σ_{sca} of the germanium cylinder (see inset) as a function of the aspect ratio r/h and frequency ω. We change the height h of the cylinder to vary the aspect ratio. The radius r of the cylinder is fixed as $r = 1030$ nm. The modes in the right resonance branch represent qBICs formed by the Friedrich-Wintgen mechanism. (**B**) The quality factor Q of the qBICs in (A) in the right branch as a function of the aspect ratio r/h.

In this section, we discuss the qBIC formation mechanism of finite Ge cylinders. This mechanism was first discussed in (*49*). We plot the rotationally averaged normalized scattering cross section σ_{sca} of a Ge cylinder as a function of its aspect ratio r/h and frequency ω in Fig. S5(A). Here, we change h to vary the aspect ratio. The radius r of the cylinder is fixed to $r = 1030$ nm (as in the

main text). In Fig. S5(A), we observe the emergence of two resonance branches. These branches represent hybrid modes formed by the interaction of Mie-type modes with indices $m = 0, n = 1$ and neighboring Mie-type modes with indices $m = 0, n = 2$. In particular, the far fields of the hybrid modes in the right branch are formed by a destructive interference of the far fields of the parent Mie modes. Due to the destructive interference, these modes possess relatively high Q-factors. In fact, these modes are qBICs formed by the Friedrich-Wintgen mechanism.

The Q-factors of these qBIC modes in the right branch are plotted in Fig. S5(B). We observe that the maximum Q-factor is achieved for the aspect ratio $r/h \approx 0.703$. In the main text, we use Ge cylinders with $r/h \approx 0.703$ that host a qBIC with $Q \approx 500$. Note that in the limit of vanishing material losses and diverging real part of the permittivity of the cylinders, the Q-factor of the qBIC diverges to infinity, forming a true BIC.

It is important to mention that the qBIC remains robust against thermal expansion and thermo-optic effects that arise due to optical pumping of Ge. We operate in a frequency regime where Ge has negligible material losses. At the considered pump intensities and using pulsed excitation, the temperature increase ΔT of the Ge cylinders remains below 1 K. From Fig. S5, we find that the Q-factor of the qBIC depends on the aspect ratio r/h rather than on the absolute values of r and h. Therefore, in the case of isotropic thermal expansion, the aspect ratio and hence the Q-factor of the qBIC is preserved. On the other hand, for anisotropic thermal expansion, using the thermal expansion coefficient of Ge ($6 \times 10^{-6}\,\mathrm{K}^{-1}$), we find that the maximum relative change in r and h is about $6 \times 10^{-4}\%$. Therefore, the aspect ratio r/h can not change by more than $10^{-3}\%$. For such small changes in r/h, the high Q-factor of the qBIC remains preserved (see Fig. S5(B)).

To assess the impact of the thermo-optic effect, we note that the Q-factor of the qBIC of the cylinders scales as $Q \sim \tilde{n}_\mathrm{r}^{1.6}$ (*49*), where $\tilde{n}_\mathrm{r}$ is the real part of the refractive index of Ge. Using the thermo-optic coefficient of Ge ($4 \times 10^{-4}\,\mathrm{K}^{-1}$), at the considered pump intensities, $\tilde{n}_\mathrm{r}$ changes by about 0.01%. This variation in $\tilde{n}_\mathrm{r}$ changes the Q-factor of the qBIC by less than 0.02%. Therefore, we conclude that the thermo-optic effect does not degrade the Q-factor of qBIC by an appreciable amount.

S6 PTCs based on metasurfaces made from homogeneous germanium spheres

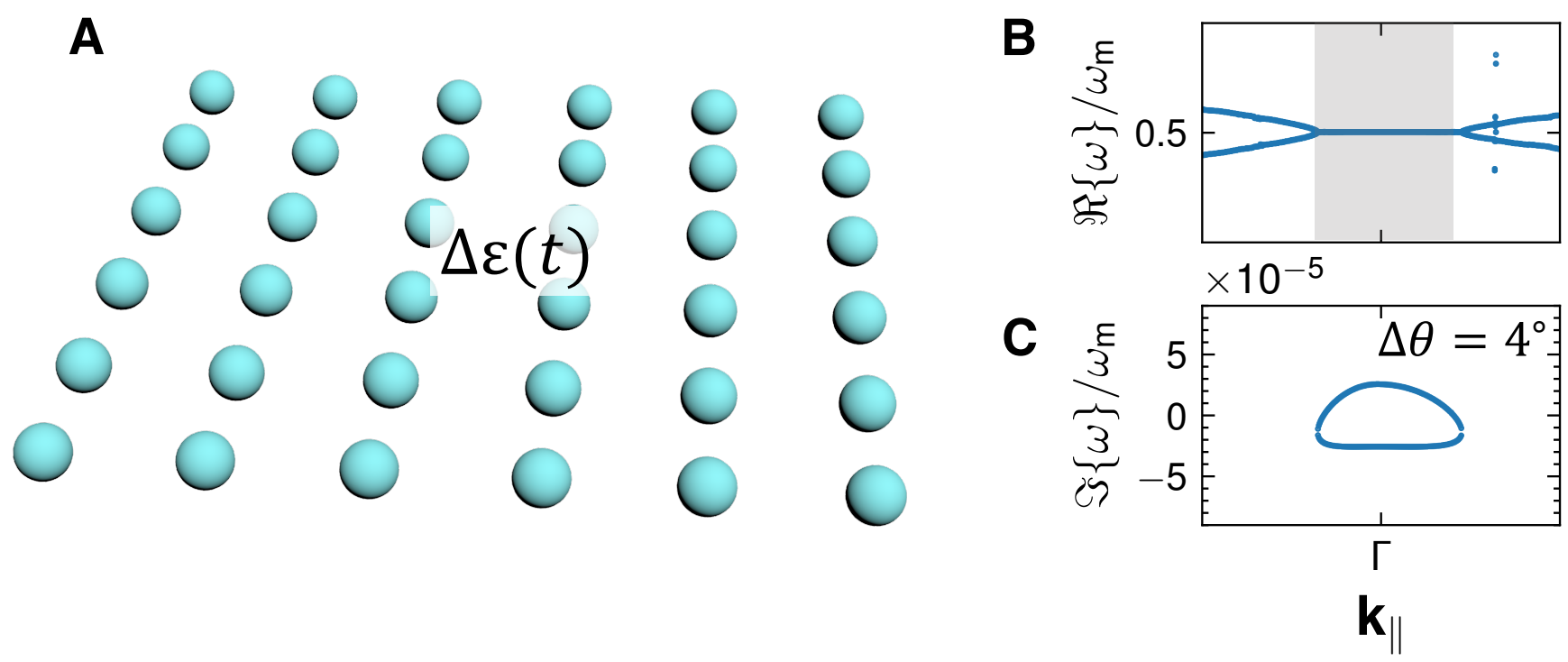

Figure S6: **PTC assisted by dipolar Mie resonances**. (**A**) A metasurface-based PTC made from a square lattice of homogeneous spheres supporting the magnetic dipolar Mie resonance. (**B**) The band structure of the time-varying metasurface made from Mie resonant homogeneous spheres illustrating a finite bandgap that corresponds to $\Delta\theta = 4°$. The gap size is identical to that obtained for qBIC resonant cylinder metasurfaces in Fig. 4(D). Here, $m = 1.4 \times 10^{-3}$ is used, which is an order of magnitude higher than that needed by the cylinder metasurface to open the same-sized bandgap. (**C**) The corresponding imaginary part of the frequency inside the bandgap, shown in (B), for a fixed $\Re(\omega)/\omega_{\mathrm{m}} = 0.5$. The displayed mode branch has a symmetry compatible with that of p-polarized plane waves. We use the lattice period $\Lambda = 3.5R_0$ for the computations. Here, R_0 is the radius of the homogeneous sphere.

A recent study showed that using metasurfaces made from low-loss homogeneous spheres reduces the required modulation amplitude to attain noticeable momentum bandgaps (*43*). The study exploits magnetic dipolar resonances of the spheres. We compare our results shown in Figs. 4(D)–(E) for the qBIC resonant cylinder metasurface to those reported in (*43*).

For a fair comparison, we consider a metasurface composed of a square lattice of homogeneous Ge spheres (see Fig. S6(A)). The radius R_0 of the spheres is optimized such that the flat bands of the metasurface due to the magnetic dipolar resonance occur at the frequency $\omega = 2\pi \times 67.3$ THz. Here, the frequency of the flat bands of the spheres is close to that of the cylinders. This choice ensures that the homogeneous sphere metasurface experiences a similar material dispersion as that

of the cylinder metasurface at the spectral location of the momentum bandgap. Therefore, we use $\omega_\mathrm{m} = 2\pi \times 134.6$ THz. The optimized radius of the spheres is $R_0 = 550$ nm. Furthermore, the quality factor of the magnetic dipolar resonance of the optimized homogeneous static sphere is $Q \approx 15$.

We plot the band structure of the time-varying metasurface in Fig. S6(B). Moreover, we also show the imaginary part of the frequency inside the momentum bandgap (see Fig. S6(C)). We find that to open a bandgap of size $\Delta\theta = 4°$, a modulation amplitude of $m = 1.4 \times 10^{-3}$ is required. This value of m corresponds to the pump intensity of 9.8 GW/cm^2, which is too close to the damage threshold of structured Ge using multi-shot pulses (*44*). On the other hand, to open the same-sized bandgap for the metasurface with cylinders, $m = 10^{-4}$ is needed, which is an order of magnitude below the damage threshold of structured Ge. Furthermore, the cylinder-based geometry is much easier to fabricate compared with the sphere-based metasurface.

S7 Link between the T-matrix and S-matrix formalism

The S-matrix $\hat{\mathbf{S}}_0$ of an isolated time-varying scatterer can be calculated from its T-matrix $\hat{\mathbf{T}}_0$ as (*77*)

$$\hat{\mathbf{S}}_0 = \hat{\mathbf{I}} + 2\hat{\mathbf{T}}_0 \,. \tag{S14}$$

Furthermore, for a metasurface made from such scatterers with the effective T-matrix $\hat{\mathbf{T}}_\mathrm{eff}$, the corresponding S-matrix $\hat{\mathbf{S}}$ is given by

$$\hat{\mathbf{S}} = \hat{\mathbf{I}} + \hat{\mathbf{V}}\,\hat{\mathbf{T}}_\mathrm{eff}\,\hat{\mathbf{U}} \,. \tag{S15}$$

Here, $\hat{\mathbf{V}}$ is the coordinate transformation matrix of the scattered field from the spherical wave to the plane wave basis, and $\hat{\mathbf{U}}$ is the coordinate transformation matrix of the incident field from the plane wave to the spherical wave basis (*57*).

S8 Details of the convergence parameters used during simulations

For performing the simulations throughout the article, we used various convergence parameters. These parameters are the maximum multipolar order l_{max}, and the total number of scattered frequencies J. Here, we list the numerical values of these parameters.

Figures 2(A),(C),(D): $l_{max} = 4$, and $J = 1$.

Figures 2(E)–(H): $l_{max} = 4$, and $J = 7$.

Figures 3(A)–(B): $l_{max} = 4$, and $J = 7$.

Figures 4(B)–(C): $l_{cut} = 10$, $l_{max} = 6$, and $J = 1$.

Figures 4(D)–(F): $l_{cut} = 10$, $l_{max} = 6$, and $J = 7$.

Figures S2(A)–(B): $l_{max} = 4$, and $J = 1$.

Figures S2(C)–(E): $l_{max} = 4$, and $J = 7$.

Figures S3(A): $l_{max} = 4$, and $J = 1$.

Figures S3(B)–(D): $l_{max} = 4$, and $J = 7$.

Figures S4(A): $l_{cut} = 6$, $l_{max} = 4$, and $J = 1$.

Figures S4(B)–(D): $l_{cut} = 6$, $l_{max} = 4$, and $J = 7$.

Figures S5(A)–(B): $l_{cut} = 10$, $l_{max} = 6$, and $J = 1$.

Figures S6(B)–(C): $l_{max} = 4$, and $J = 7$.

REFERENCES

1. E. Galiffi, R. Tirole, S. Yin, H. Li, S. Vezzoli, P. Huidobro, M. Silveirinha, R. Sapienza, A. Alù, J. Pendry, Photonics of time-varying media. *Adv. Photonics* **4**, 014002 (2022).

2. Z. Hayran, F. Monticone, Capturing broadband light in a compact bound state in the continuum. *ACS Photonics* **8**, 813–823 (2021).

3. Z. Yang, M. Liu, D. Smirnova, A. Komar, M. Shcherbakov, T. Pertsch, D. Neshev, Ultrafast Q-boosting in semiconductor metasurfaces. *Nanophotonics* **13**, 2173–2182 (2024).

4. O. Schiller, Y. Plotnik, O. Segal, M. Lyubarov, M. Segev, Time-domain bound states in the continuum. *Phys. Rev. Lett.* **133**, 263802 (2024).

5. N. Engheta, Four-dimensional optics using time-varying metamaterials. *Science* **379**, 1190–1191 (2023).

6. N. Engheta, Metamaterials with high degrees of freedom: Space, time, and more. *Nanophotonics* **10**, 639–642 (2021).

7. I. Liberal, A. Manjavacas, Synthetic crystal rotation with spacetime metamaterials. *Phys. Rev. Lett.* **136**, 146903 (2026).

8. F. R. Prudêncio, M. G. Silveirinha, Synthetic axion response with space-time crystals. *Phys. Rev. Appl.* **19**, 024031 (2023).

9. P. Huidobro, M. Silveirinha, E. Galiffi, J. Pendry, Homogenization theory of space-time metamaterials. *Phys. Rev. Appl.* **16**, 014044 (2021).

10. E. Galiffi, A. C. Harwood, S. Vezzoli, R. Tirole, A. Alù, R. Sapienza, Optical coherent perfect absorption and amplification in a time-varying medium. *Nat. Photonics* **20**, 163–169 (2026).

11. S. Koufidis, T. Koutserimpas, F. Monticone, M. Mccall, Electromagnetic wave propagation in time-periodic chiral media. *Opt. Mater. Express* **14**, 3006–3029 (2024).

12. V. Pacheco-Peña, N. Engheta, Antireflection temporal coatings. *Optica* **7**, 323–331 (2020).

13. M. S. Mirmoosa, T. Setälä, A. Norrman, Quantum state engineering and photon statistics at electromagnetic time interfaces. *Phys. Rev. Res.* **7**, 013120 (2025).

14. E. Cassedy, Temporal instabilities in traveling-wave parametric amplifiers (correspondence). *IRE Trans. Microwave Theory Tech.* **10**, 86–87 (1962).

15. J. R. Zurita-Sánchez, P. Halevi, J. C. Cervantes-González, Reflection and transmission of a wave incident on a slab with a time-periodic dielectric function $\varepsilon(t)$. *Phys. Rev. A* **79**, 053821 (2009).

16. M. M. Asgari, P. Garg, X. Wang, M. S. Mirmoosa, C. Rockstuhl, V. Asadchy, Theory and applications of photonic time crystals: A tutorial. *Adv. Opt. Photonics* **16**, 958–1063 (2024).

17. T. T. Koutserimpas, Parametric amplification interactions in time-periodic media: Coupled waves theory. *J. Opt. Soc. Am. B* **39**, 481–489 (2022).

18. C. Rizza, G. Castaldi, V. Galdi, Nonlocal effects in temporal metamaterials. *Nanophotonics* **11**, 1285–1295 (2022).

19. D. Ramaccia, D. L. Sounas, A. Alù, A. Toscano, F. Bilotti, Phase-induced frequency conversion and doppler effect with time-modulated metasurfaces. *IEEE Trans. Antennas Propag.* **68**, 1607–1617 (2020).

20. S. Zhang, J. Dong, H. Li, J. Xu, B. Shapiro, Longitudinal optical phonons in photonic time crystals containing a stationary charge. *Phys. Rev. B* **110**, L100306 (2024).

21. S. John, Strong localization of photons in certain disordered dielectric superlattices. *Phys. Rev. Lett.* **58**, 2486 (1987).

22. E. Yablonovitch, Inhibited spontaneous emission in solid-state physics and electronics. *Phys. Rev. Lett.* **58**, 2059 (1987).

23. M. Lyubarov, Y. Lumer, A. Dikopoltsev, E. Lustig, Y. Sharabi, M. Segev, Amplified emission and lasing in photonic time crystals. *Science* **377**, 425–428 (2022).

24. J. Park, K. Lee, R.-Y. Zhang, H.-C. Park, J.-W. Ryu, G. Y. Cho, M. Y. Lee, Z. Zhang, N. Park, W. Jeon, J. Shin, C. T. Chan, B. Min, Spontaneous emission decay and excitation in photonic time crystals. *Phys. Rev. Lett.* **135**, 133801 (2025).

25. A. Dikopoltsev, Y. Sharabi, M. Lyubarov, Y. Lumer, S. Tsesses, E. Lustig, I. Kaminer, M. Segev, Light emission by free electrons in photonic time-crystals. *Proc. Natl. Acad. Sci. U.S.A.* **119**, e2119705119 (2022).

26. R. Carminati, H. Chen, R. Pierrat, B. Shapiro, Universal statistics of waves in a random time-varying medium. *Phys. Rev. Lett.* **127**, 094101 (2021).

27. Y. Sharabi, E. Lustig, M. Segev, Disordered photonic time crystals. *Phys. Rev. Lett.* **126**, 163902 (2021).

28. B. Apffel, S. Wildeman, A. Eddi, E. Fort, Experimental implementation of wave propagation in disordered time-varying media. *Phys. Rev. Lett.* **128**, 094503 (2022).

29. M. Coppolaro, M. Moccia, G. Castaldi, V. Galdi, Exploring aperiodic order in photonic time crystals. *Phys. Rev. Appl.* **24**, L011001 (2025).

30. J. R. Reyes-Ayona, P. Halevi, Observation of genuine wave vector (k or β) gap in a dynamic transmission line and temporal photonic crystals. *Appl. Phys. Lett.* **107**, 074101 (2015).

31. J. Park, H. Cho, S. Lee, K. Lee, K. Lee, H. C. Park, J.-W. Ryu, N. Park, S. Jeon, B. Min, Revealing non-Hermitian band structure of photonic Floquet media. *Sci. Adv.* **8**, eabo6220 (2022).

32. X. Wang, M. S. Mirmoosa, V. S. Asadchy, C. Rockstuhl, S. Fan, S. A. Tretyakov, Metasurface-based realization of photonic time crystals. *Sci. Adv.* **9**, eadg7541 (2023).

33. Z. Hayran, J. B. Khurgin, F. Monticone, $\hbar\omega$ versus $\hbar k$: Dispersion and energy constraints on time-varying photonic materials and time crystals. *Opt. Mater. Express* **12**, 3904–3917 (2022).

34. M. Z. Alam, I. D. Leon, R. W. Boyd, Large optical nonlinearity of indium tin oxide in its epsilon-near-zero region. *Science* **352**, 795–797 (2016).

35. J. Bohn, T. S. Luk, S. Horsley, E. Hendry, Spatiotemporal refraction of light in an epsilon-near-zero indium tin oxide layer: Frequency shifting effects arising from interfaces. *Optica* **8**, 1532–1537 (2021).

36. R. Tirole, S. Vezzoli, E. Galiffi, I. Robertson, D. Maurice, B. Tilmann, S. A. Maier, J. B. Pendry, R. Sapienza, Double-slit time diffraction at optical frequencies. *Nat. Phys.* **19**, 999–1002 (2023).

37. Y. Zhou, M. Z. Alam, M. Karimi, J. Upham, O. Reshef, C. Liu, A. E. Willner, R. W. Boyd, Broadband frequency translation through time refraction in an epsilon-near-zero material. *Nat. Commun.* **11**, 2180 (2020).

38. W. Jaffray, S. Stengel, F. Biancalana, C. B. Fruhling, M. Ozlu, M. Scalora, A. Boltasseva, V. M. Shalaev, M. Ferrera, Spatio-spectral optical fission in time-varying subwavelength layers. *Nat. Photonics* **19**, 558–566 (2025).

39. E. Lustig, O. Segal, S. Saha, E. Bordo, S. N. Chowdhury, Y. Sharabi, A. Fleischer, A. Boltasseva, O. Cohen, V. M. Shalaev, M. Segev, Time-refraction optics with single cycle modulation. *Nanophotonics* **12**, 2221–2230 (2023).

40. J. B. Khurgin, Photonic time crystals and parametric amplification: Similarity and distinction. *ACS Photonics* **11**, 2150–2159 (2024).

41. N. K. Hon, R. Soref, B. Jalali, The third-order nonlinear optical coefficients of Si, Ge, and $Si_{1-x}Ge_x$ in the midwave and longwave infrared. *J. Appl. Phys.* **110**, 011301 (2011).

42. B. Borchers, C. Brée, S. Birkholz, A. Demircan, G. Steinmeyer, Saturation of the all-optical Kerr effect in solids. *Opt. Lett.* **37**, 1541–1543 (2012).

43. X. Wang, P. Garg, M. S. Mirmoosa, A. G. Lamprianidis, C. Rockstuhl, V. S. Asadchy, Expanding momentum bandgaps in photonic time crystals through resonances. *Nat. Photonics* **19**, 149–155 (2025).

44. R. Malik, B. Mills, J. H. V. Price, M. Petrovich, Z. Moktadir, Z. Li, H. N. Rutt, Determination of the mid-IR femtosecond surface-damage threshold of germanium. *Appl. Phys. A* **113**, 127–133 (2013).

45. D. R. Austin, K. R. P. Kafka, Y. H. Lai, Z. Wang, C. I. Blaga, E. A. Chowdhury, Femtosecond laser damage of germanium from near- to mid-infrared wavelengths. *Opt. Lett.* **43**, 3702–3705 (2018).

46. C. Kern, M. Zürch, J. Petschulat, T. Pertsch, B. Kley, T. Käsebier, U. Hübner, C. Spielmann, Comparison of femtosecond laser-induced damage on unstructured vs. nano-structured Au-targets. *Appl. Phys. A* **104**, 15–21 (2011).

47. G. Grinblat, Y. Li, M. P. Nielsen, R. F. Oulton, S. A. Maier, Efficient third harmonic generation and nonlinear subwavelength imaging at a higher-order anapole mode in a single germanium nanodisk. *ACS Nano* **11**, 953–960 (2017).

48. C. W. Hsu, B. Zhen, A. D. Stone, J. D. Joannopoulos, M. Soljačić, Bound states in the continuum. *Nat. Rev. Mater.* **1**, 16048 (2016).

49. M. V. Rybin, K. L. Koshelev, Z. F. Sadrieva, K. B. Samusev, A. A. Bogdanov, M. F. Limonov, Y. S. Kivshar, High-*Q* supercavity modes in subwavelength dielectric resonators. *Phys. Rev. Lett.* **119**, 243901 (2017).

50. K. Koshelev, S. Kruk, E. Melik-Gaykazyan, J.-H. Choi, A. Bogdanov, H.-G. Park, Y. Kivshar, Subwavelength dielectric resonators for nonlinear nanophotonics. *Science* **367**, 288–292 (2020).

51. A. A. Bogdanov, K. L. Koshelev, P. V. Kapitanova, M. V. Rybin, S. A. Gladyshev, Z. F. Sadrieva, K. B. Samusev, Y. S. Kivshar, M. F. Limonov, Bound states in the continuum and Fano resonances in the strong mode coupling regime. *Adv. Photonics* **1**, 016001 (2019).

52. P. C. Waterman, Matrix formulation of electromagnetic scattering. *Proc. IEEE* **53**, 805–812 (1965).

53. Y. Xiao, *Propagation of Optical Pulses in Dynamic Media: A Time Transformation Method* (University of Rochester, 2014).

54. F. Monticone, A. Alù, Embedded photonic eigenvalues in 3D nanostructures. *Phys. Rev. Lett.* **112**, 213903 (2014).

55. L. Caspani, R. P. M. Kaipurath, M. Clerici, M. Ferrera, T. Roger, J. Kim, N. Kinsey, M. Pietrzyk, A. Di Falco, V. M. Shalaev, A. Boltasseva, D. Faccio, Enhanced nonlinear refractive index in ϵ-near-zero materials. *Phys. Rev. Lett.* **116**, 233901 (2016).

56. M. G. Silveirinha, Trapping light in open plasmonic nanostructures. *Phys. Rev. A* **89**, 023813 (2014).

57. P. Garg, A. G. Lamprianidis, D. Beutel, T. Karamanos, B. Verfürth, C. Rockstuhl, Modeling four-dimensional metamaterials: A T-matrix approach to describe time-varying metasurfaces. *Opt. Express* **30**, 45832–45847 (2022).

58. P. Kelly, W. Zhang, M. Liu, L. Kuznetsova, Engineering the structural, plasmonic, and optical properties of multilayered aluminum-doped zinc oxide metamaterial grown by pulsed laser deposition. *Appl. Opt.* **58**, 5681–5686 (2019).

59. T. Amotchkina, M. Trubetskov, D. Hahner, V. Pervak, Characterization of e-beam evaporated Ge, YbF3, ZnS, and LaF3 thin films for laser-oriented coatings. *Appl. Opt.* **59**, A40–A47 (2020).

60. G. Gantzounis, Plasmon modes of axisymmetric metallic nanoparticles: A group theory analysis. *J. Phys. Chem. C* **113**, 21560–21565 (2009).

61. E. Panagiotidis, E. Almpanis, N. Papanikolaou, N. Stefanou, Optical transitions and nonreciprocity in spatio-temporally periodic layers of spherical particles. *Adv. Opt. Mater.* **11**, 2202812 (2023).

62. P. C. Waterman, Matrix theory of elastic wave scattering. *J. Acoust. Soc. Am.* **60**, 567–580 (1976).

63. D. Beutel, A. Groner, C. Rockstuhl, I. Fernandez-Corbaton, Efficient simulation of biperiodic, layered structures based on the T-matrix method. *J. Opt. Soc. Am. B* **38**, 1782–1791 (2021).

64. D. Beutel, I. Fernandez-Corbaton, C. Rockstuhl, treams–A *T*-matrix-based scattering code for nanophotonics. *Comput. Phys. Commun.* **297**, 109076 (2024).

65. A. G. Lamprianidis, "Generalized transition matrix methods for the analysis of linear nanophotonic systems," thesis, Karlsruher Institut für Technologie (KIT) (2023).

66. G. Ptitcyn, A. Lamprianidis, T. Karamanos, V. Asadchy, R. Alaee, M. Müller, M. Albooyeh, M. S. Mirmoosa, S. Fan, S. Tretyakov, C. Rockstuhl, Floquet-mie theory for time-varying dispersive spheres. *Laser Photonics Rev.* **17**, 2100683 (2023).

67. N. Stefanou, I. Stefanou, E. Almpanis, N. Papanikolaou, P. Garg, C. Rockstuhl, Light scattering by a periodically time-modulated object of arbitrary shape: The extended boundary condition method. *J. Opt. Soc. Am. B* **40**, 2842–2850 (2023).

68. F. Olver, A. O. Daalhuis, D. Lozier, B. Schneider, R. Boisvert, C. Clark, B. Miller, B. Saunders, H. Cohl, M. McClain, Eds., *NIST Digital Library of Mathematical Functions* (National Institute of Standards and Technology, 2021); https://dlmf.nist.gov.

69. A. Rahimzadegan, T. Karamanos, R. Alaee, A. Lamprianidis, D. Beutel, R. Boyd, C. Rockstuhl, A comprehensive multipolar theory for periodic metasurfaces. *Adv. Opt. Mater.* **10**, 2102059 (2022).

70. C. Wolff, K. Busch, N. A. Mortensen, Modal expansions in periodic photonic systems with material loss and dispersion. *Phys. Rev. B* **97**, 104203 (2018).

71. Y. Nakatsukasa, O. Sète, L. N. Trefethen, The AAA algorithm for rational approximation. *SIAM J. Sci. Comput.* **40**, A1494–A1522 (2018).

72. F. Betz, M. Hammerschmidt, L. Zschiedrich, S. Burger, F. Binkowski, Efficient rational approximation of optical response functions with the AAA algorithm. *Laser Photonics Rev.* **18**, 2400584 (2024).

73. J. D. Fischbach, F. Betz, N. Asadova, P. Tassan, D. Urbonas, T. Stöferle, R. F. Mahrt, S. Burger, C. Rockstuhl, F. Binkowski, T. J. Sturges, A framework to compute resonances arising from multiple scattering. *Adv. Theor. Simul.* **8**, 2400989 (2025).

74. J. B. Khurgin, M. Clerici, N. Kinsey, Fast and slow nonlinearities in epsilon-near-zero materials. *Laser Photonics Rev.* **15**, 2000291 (2021).

75. V. Asadchy, A. Lamprianidis, G. Ptitcyn, M. Albooyeh, R. Rituraj, T. Karamanos, S. Alaee, C. Tretyakov, S. F. Rockstuhl, Parametric mie resonances and directional amplification in time-modulated scatterers. *Phys. Rev. Appl.* **18**, 054065 (2022).

76. N. Ustimenko, C. Rockstuhl, A. B. Evlyukhin, Resonances in finite-size all-dielectric metasurfaces for light trapping and propagation control. *Phys. Rev. B* **109**, 115436 (2024).

77. P. C. Waterman, *T*-matrix methods in acoustic scattering. *J. Acoust. Soc. Am.* **125**, 42–51 (2009).
